# Rationale and protocol of the ENGAGE study: a double-blind randomized controlled preference trial using a comprehensive cohort design to measure the effect of a cognitive and leisure-based intervention in older adults with a memory complaint

**DOI:** 10.1186/s13063-019-3250-6

**Published:** 2019-05-22

**Authors:** S. Belleville, A. Moussard, A. I. Ansaldo, P. Belchior, L. Bherer, N. Bier, V. D. Bohbot, M.-A. Bruneau, L. L. Cuddy, B. Gilbert, R. Jokel, K. Mahalingam, K. McGilton, K. J. Murphy, G. Naglie, E. Rochon, A. K. Troyer, N. D. Anderson

**Affiliations:** 1 Université de Montréal, Montreal, Canada; 2 Research Center, Institut Universitaire de Gériatrie de Montréal, Montreal, Canada; 3 University of Toronto, Toronto, Canada; 4 Baycrest Health Sciences, Toronto, Canada; 5 McGill University, Montreal, Canada; 6 Douglas Mental Health University Institute, Montreal, Canada; 7 Queen’s University, Kingston, Canada; 8 Toronto Rehabilitation Institute - the University Health Network, Toronto, Canada

**Keywords:** Cognitive training, Cognitive intervention, Stimulating leisure activities, Cognitive reserve, Design, Preference trial, Dementia prevention, Subjective cognitive decline, Early mild cognitive impairment

## Abstract

**Background:**

Leisure activities can be both enjoyable and cognitively stimulating, and participation in such activities has been associated with reduced age-related cognitive decline. Thus, integrating stimulating leisure activities in cognitive training programs may represent a powerful and innovative approach to promote cognition in older adults at risk of dementia. The ENGAGE study is a randomized controlled, double-blind preference trial with a comprehensive cohort design that will test the efficacy and long-term impact of an intervention that combines cognitive training and cognitively stimulating leisure activities.

**Methods:**

One hundred and forty-four older adults with a memory complaint will be recruited in Montreal and Toronto. A particular effort will be made to reach persons with low cognitive reserve. Participants will be randomly assigned to one of two conditions: cognitive + leisure training (ENGAGE-MUSIC/SPANISH) or active control (ENGAGE-DISCOVERY). The ENGAGE-MUSIC/SPANISH training will include teaching of mnemonic and attentional control strategies, casual videogames selected to train attention, and classes in music or Spanish as a second language. The ENGAGE-DISCOVERY condition will comprise psychoeducation on cognition and the brain, low-stimulating casual videogames and documentary viewing with discussions. To retain the leisure aspect of the activities, participants will be allowed to exclude either music or Spanish at study entry if they strongly dislike one of these activities. Participants randomized to ENGAGE-MUSIC/SPANISH who did not exclude any activity will be assigned to music or Spanish based on a second random assignment. Training will be provided in 24 2-h sessions over 4 months. Outcomes will be measured at baseline, at 4-month follow-up, and at 24-month follow-up. The primary outcome will be cognitive performance on a composite measure of episodic memory (delayed recall scores for words and face-name associations) measured at baseline and at the 4-month follow-up. Secondary outcomes will include a composite measure of attention (speed of processing, inhibition, dual tasking, and shifting), psychological health, activities of daily living, and brain structure and function and long-term maintenance measured at the 24-month follow-up. Information on cognitive reserve proxies (education and lifestyle questionnaires), sex and genotype (apolipoprotein (Apo)E4, brain-derived neurotrophic factor (BDNF), and catechol-O-methyltransferase (COMT)) will be collected and considered as moderators of training efficacy.

**Discussion:**

This study will test whether a program combining cognitive training with stimulating leisure activities can increase cognition and reduce cognitive decline in persons at risk of dementia.

**Trial registration:**

NCT03271190. Registered on 5 September 2017.

**Electronic supplementary material:**

The online version of this article (10.1186/s13063-019-3250-6) contains supplementary material, which is available to authorized users.

## Background

Approximately 19% of Alzheimer’s disease (AD) cases worldwide have been attributed to cognitive inactivity; thus, increasing cognitive activity may have a major impact on the number of cases of AD [1]. This is in line with the concept of cognitive reserve, which proposes that some individuals are more resistant to age-related brain decline or diseases partly due to a positive effect of lifelong cognitive engagement (e.g., education attainment, occupation, hobbies, social network) on brain structure and function [2–5]. These findings have significant implications for the development of strategies to reduce cognitive decline in aging and they have motivated the development of nonpharmacological approaches which include cognitive training or activity engagement as one of the key ingredients. Cognitive training refers to a range of formal techniques that are designed to improve cognitive processes, whereas activity engagement refers to leisure or volunteering activities that are selected to be cognitively stimulating.

Cognitive training is a form of late-life stimulation and has been widely used in studies meant to increase the cognition of older adults. Empirical studies have shown that different types of cognitive training increase cognitive abilities in healthy older adults [6, 7] or persons with mild cognitive impairment (MCI) [8–10]. However, limitations to the impact of cognitive training may come from the fact that these programs are sometimes perceived as lacking meaningfulness, hence limiting the engagement of older adults. This might be particularly true for persons with a lower educational or socioeconomic status [11, 12]. Leisure activities, by contrast, are perceived to be enjoyable and often accessible. Leisure activities may also be neuroprotective since engaging in cognitively stimulating leisure activities in later life has been reported to protect against age-related cognitive decline and dementia [4, 5]. Incorporating leisure activities into cognitively stimulating programs might thus have tremendous potential because they may make interventions more enjoyable and meaningful to older adults and because they are relatively easy to implement in the community [13, 14].

The ENGAGE study was designed to develop and test an innovative leisure-based intervention to enhance cognition and reduce cognitive decline over time. The program includes strategy-based memory training and formal attention training, carefully selected casual videogames, and either music (ENGAGE-MUSIC) or Spanish (ENGAGE-SPANISH) lessons. Selecting the most appropriate leisure activities was done on the basis of empirical findings showing that learning a second language [15], receiving musical training [16–18], or playing certain types of casual videogames [19] has a positive effect on cognitive and brain aging. The term ‘casual videogames’ refers here to commercially available leisure platform videogames that were not designed to improve cognition per se, but which may have the potential to increase attention or speed processing.

The study targets older adults with a subjective cognitive complaint but no dementia, referred to as subjective cognitive decline (SCD). People with SCD express concern about their memory and are at a higher risk of progressing to dementia than older adults with no memory complaints [20, 21]. Those individuals might benefit tremendously from cognitive training because they may be highly motivated to improve their cognition and they retain the cognitive capacities to learn new strategies and apply them to their daily life. Furthermore, if they are in a very early phase of a neurodegenerative process, they may be more amenable to training-induced brain plasticity than in later stages of a neurodegenerative disease [22, 23]. A particular effort will be made to recruit individuals with low cognitive reserve by including individuals with lower levels of formal education. It is critical that representative samples be recruited in intervention studies because different populations might respond to different types of intervention. For instance, interventions designed to increase cognition might be particularly beneficial to persons with lower education as they are more at risk of developing dementia [1].

This study will be the first to test the efficacy of an intervention that combines leisure-based and formal cognitive training approaches. It will also address important knowledge gaps regarding cognition-focused interventions in the current scientific literature. The first critical issue is the lack of evidence that cognitive training improves activities of daily life [24]. Indeed, most training studies have failed to measure transfer or reported limited context transfer, defined as cognitive improvement carried over into an environment different from the one where training occurred [25, 26]. The lack of context transfer might be due to the fact that participants have difficulties applying the newly learned strategies from the classroom context to more complex situations encountered in their daily life. Notably, context transfer can be improved by training participants to apply the learned strategies in varied and more ecologically valid situations [12]. Thus, an additional advantage for including leisure activities is that they provide the opportunity to apply the different strategies learned during formal cognitive training to situations encountered during the music and Spanish sessions, and while playing videogames. For example, semantic association strategies will be used to learn Spanish vocabulary. Thus, the leisure sessions will offer an ecologically valid setting for participants to use and practice their strategic skills. It is anticipated that reinforcing and broadening strategy use will make participants more likely to employ them in other contexts within their everyday life. Another innovative aspect of the study will be to investigate individual differences in response to cognitive training. Very little is known about the characteristics that identify who will benefit the most from an intervention. The study will analyze the effect of a small set of variables (education, age, sex) that may be associated with differences in gains from training based on prior findings. As participants will be deeply phenotyped, it will also be possible to assess the effects of other variables on an exploratory basis (e.g., baseline brain structure and function, health). The study will also measure the effect that cognitive training has on brain structure and function. A few studies have shown that cognitive training in persons with MCI may lead to greater brain activation and increased structural connectivity [27, 28]. However, researchers still have a very limited understanding of how these interventions alter brain structure and function in older adults, whether these effects are long-lasting, whether the characteristics of participants contribute to these brain changes, how structural and functional changes relate with one another, and whether training gains depend on specialized brain regions or involve neurocompensatory responses from alternative regions.

Finally, previous studies have been limited by a number of methodological flaws that will be controlled for in the present study. In particular, many previous studies have used a no-contact group as a control condition and/or have failed to account for expectation effects (i.e., whether participants expect cognitive changes from the training program). This is a major problem since expectation has been found to be a strong predictor of efficacy in cognitive training studies [29]. Thus, a failure to account for expectation, or the inclusion of an inappropriate control condition, can produce group differences that are not genuinely related to the content of the intervention. To reduce a potential effect of expectation, care will be taken to ensure that the active control intervention is not perceived as inactive by participants. This should increase their expectation that the intervention they receive might improve their cognition, hence controlling for an expectation difference between the control and experimental group. Furthermore, expectations will be measured and controlled for if they are found to differ among groups. Lastly, a 24-month follow-up assessment will evaluate the long-term effects of the ENGAGE program, since little is known about the durability of the benefits of cognitive training and leisure-based interventions.

### Objectives and hypotheses

The primary objective of this study is to assess whether the ENGAGE-MUSIC/SPANISH program leads to cognitive gains in episodic memory at the immediate follow-up assessment in older adults with a subjective memory complaint when compared with an active control condition. The secondary objectives of this study are: to assess whether these cognitive gains are maintained at the 24-month follow-up; to determine the effect of the program on attentional control, psychological health, activities of daily living, and brain structure and function as measured by magnetic resonance imaging (MRI); and to identify if the characteristics of participants modulate outcomes. Finally, the study will examine whether music and Spanish lessons produce different effects on the primary and secondary outcome measures.

It is expected that the ENGAGE-MUSIC/SPANISH training will show evidence of efficacy: persons enrolled in the ENGAGE-MUSIC/SPANISH training program will have larger gains in the memory composite measure following training than participants enrolled in the active control condition. Secondary hypotheses involve maintenance of training gains, and effects of training on attentional control, daily functioning, well-being, and brain structure and function. It is hypothesized that training effects on the memory composite will be maintained at the 24-month follow-up assessment. As the ENGAGE-MUSIC/SPANISH program includes a component of attention training, it is expected that measures of attentional control will improve. It is also expected that cognitive improvement will transfer to measures of activities of daily living (e.g., managing finances, medication, grocery shopping) and that it will increase indicators of psychological health. Also, the ENGAGE-MUSIC/SPANISH training will result in measurable effects on the brain as assessed by both structural (cortical thickness and regional grey matter volume) and task-related MRI activation, and that these changes will be correlated with improved cognition. Finally, we hypothesize that participant characteristics (sex, age, education) will modulate efficacy and transfer.

## Methods

The ENGAGE study is registered with the US National Institutes of Health clinical trials registry (ClinicalTrials.gov identifier NCT03271190). This trial report complies with the Standard Protocol Items: Recommendations for Interventional Trials (SPIRIT) statement (see Additional file 1 for the SPIRIT checklist).

### Design

**Fig. 1:**
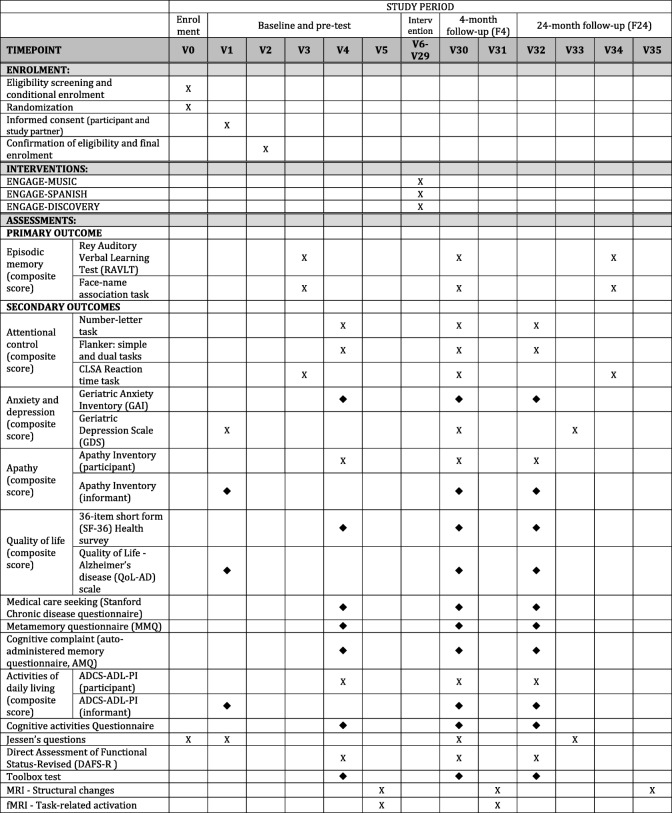
Illustration of the study design. *ADCS-ADL-PI* Alzheimer’s Disease Cooperative Study—Activities of daily living—Prevention Instrument; *CLSA* Canadian Longitudinal Study on Aging; *(f)MRI* (functional) magnetic resonance imaging; *V0* Phone screening interview; *V1* First visit (screening); *V2* Second visit (clinical assessment); *V3* Third visit (neuropsychological assessment); *V4* Fourth visit (neuropsychological assessment); *V5* Fifth visit (MRI; includes fMRI for a subgroup of participants); *V6-29* ENGAGE Intervention sessions (24 visits); *V30* 4-month follow-up neuropsychological assessment; *V31* 4-month follow-up MRI/fMRI (for a subgroup of participants); *V32 & V34* 24-month follow-up neuropsychological assessment; *V33* 24-month follow-up clinical visit; *V35* 24-month follow-up MRI. X: administered during the visit; ♦: given to be completed at home by next visit

The design of the study is presented in Fig. 1. This study is a 2-year, double-blind, randomized controlled preference trial with a comprehensive cohort design [30, 31]. In the current instantiation of this preference design, participants can exclude one of the interventions, based on evidence that imposing an intervention would bias the sample by reducing participation or increasing selective drop-out which would undermine the advantage of randomization [30, 31]. Furthermore, a leisure activity should be enjoyable by definition. Allowing participants to exclude an activity that they strongly dislike is more likely to favor motivation and engagement and reproduce real-world conditions, perhaps mitigating some of the identified obstacles to real-world implementation.

Participants will be randomly assigned to one of two conditions: the intervention program combining stimulating leisure activities with formal cognitive training (ENGAGE-MUSIC/SPANISH) or an active control condition (ENGAGE-DISCOVERY). As the program is based on the notion that leisure activities are pleasant, and because some people may have strong discomfort with learning music or be reluctant to learn foreign languages, participants will have the option of excluding either the “Music” or “Spanish” activity at their entry in the study. Participants randomized in the intervention (ENGAGE SPANISH/MUSIC) arm who did not exclude one activity will receive either Spanish or music training based on a second independent random assignment. Thus, the intervention versus active control comparison is fully randomized, but secondary analyses comparing the two leisure activities will rely on a partially randomized cohort. Furthermore, and as described below, analyses will first compare the fully randomized cohorts followed by a comparison of the comprehensive cohort, including those in the preference arms.

For all three groups (ENGAGE-MUSIC, ENGAGE-SPANISH, and ENGAGE-DISCOVERY) 24 training sessions will be delivered over a 4-month period. Participants will be assessed at baseline, which will be no more than 8 weeks prior to training (PRE), at 4-month follow-up, i.e., no more than 4 weeks following training (F4), and 24 months after baseline (F24).

### Study population

This study will be carried out at two sites in Canada, in Montreal (Institut Universitaire de Gériatrie de Montréal, IUGM, of the CIUSSS du Centre-Sud-de-l'Ile-de-Montréal) and Toronto (Baycrest Health Sciences). The study is part of the Canadian Consortium on Neurodegeneration in Aging (CCNA) and participants will be enrolled in the CCNA Comprehensive Assessment of Neurodegeneration and Dementia (COMPASS-ND) cohort (please refer to ClinicalTrials.gov NCT03402919 and to Additional file 2: Appendix 1 for a more detailed description of the COMPASS-ND study). A subset of participants will be recruited from the Consortium for the Early Identification of Alzheimer’s Disease (CIMA-Q), a Quebec consortium working in partnership with the CCNA [32]. One hundred and forty-four participants will be recruited from the Montreal CIMA-Q cohort as well as from the Montreal and Toronto community through flyers, local magazines and newspapers, and talks at community centers.

#### Inclusion criteria

Included participants will be aged 60–85 years at baseline and have a memory complaint which worries them. Thus, they will answer ‘Yes’ to both of the following questions: “Do you feel like your memory is becoming worse?” and “Does this worry you?” [20, 21]. They will also have minimal or no cognitive deficit. Specifically, they will have: (1) a delayed recall score on Story A of the Logical Memory subtest of the Wechsler Memory Scale—Revised [33] above the ADNI education-adjusted cutoffs [34] (≥ 9 for 16+ years of education; ≥ 5 for 8–15 years of education; ≥ 3 for 0–7 years of education); (2) a Montreal Cognitive Assessment (MoCA) total score above 20; (3) a delayed recall score on the CERAD word list above 4; and (4) a global Clinical Dementia Rating (CDR) score lower than 1.0. Based on these criteria, the individuals recruited in the study will meet criteria for SCD or early MCI [20, 21, 35].

Additional inclusion criteria are sufficient visual and auditory acuity, sufficient English/French proficiency to undergo assessment and participate in the intervention, the ability to commit for the whole intervention and follow-up assessments, having an internet connection at home, and an informant to provide corroborative information.

#### Exclusion criteria

Exclusion criteria are as follow: the presence of disease or injury of the central nervous system, such as moderate to severe chronic static leukoencephalopathy (including previous traumatic injury), dementia, multiple sclerosis, a serious developmental handicap, subdural hematoma (past or current), subarachnoid hemorrhage (past or current), primary cerebral tumor or cerebral metastases, epilepsy (current), symptomatic stroke within the previous year, dementia or other neurodegenerative diseases, and other rare brain illnesses; history of intracranial surgery; major surgery within the past 2 months; major depression or clinical anxiety disorders; schizophrenia or other major psychiatric disorders; ongoing alcohol or drug abuse that in the opinion of the investigator may interfere with the participant’s ability to comply with the study procedures; inability to undergo an MRI scan due to medical contraindications or intolerance for the procedure; being a musician, or having more than 5 years of formal music training or having more than 10 years of choir experience; speaking Spanish, or more than 5 years of Spanish classes (beyond secondary school) or having lived in a Spanish-speaking country; being currently involved in music or Spanish classes; being currently involved in another research project; and having participated in a strategy-based memory training program in the past.

A special effort will be made to include low-education individuals who are often less represented in such studies. Recruitment strategies have been developed to access this segment of the population, including identifying lower income neighborhoods in both cities and connecting with community centers in those neighborhoods, organizing lay-audience presentations at those community centers, placing newspaper adverts targeting low-income communities, and targeting low-education individuals in participant databases.

### Procedure

Interested participants will be screened over the telephone to determine eligibility for the study including the presence of a memory complaint with worries. Participants and informants will be presented with the inform and consent form and sign it with the research nurse at the beginning of their first visit (see Additional file 2: Appendices 4 and 5). They will be invited to undergo baseline (PRE) testing that will include two visits for screening and clinical assessment to determine whether participants meet inclusion criteria for cognition (see ‘Study population’ section above), a clinical and physical examination, and biological sampling. They will then be invited to two sessions to complete the neuropsychological assessment and one MRI session (with a functional MRI (fMRI) condition for a subgroup of participants). Finally, an optional session involves a lumbar puncture. After the 24 intervention visits, participants will undergo the follow-up assessment (F4) including a neuropsychological assessment and an MRI/fMRI for a subgroup of participants. All participants will be invited for a last follow-up assessment 24 months after PRE, including clinical and neuropsychological assessment as well as an MRI (see Fig. 1).

### Randomization and blinding procedure

The opportunity to exclude either music or Spanish training will be offered to all participants prior to randomization. Randomization will be done individually (i.e., one participant at a time as they enter the study) with random lists of binary numbers. Lists will be generated using the Random.org online software. Randomization will be controlled by a person from the Montreal site who is independent of the study. A first randomization will allocate participants to the intervention or control condition by matching the sequential list of participant identification codes with the list of random condition allocation. This will be done with a 2 to 1 ratio between intervention and control conditions. A second randomization will be used to allocate participants in the intervention condition to either Spanish or music training, using a 1 to 1 ratio for participants who did not exclude either of these two activities. Participants randomized in the intervention condition who excluded music or Spanish training will be placed in their preferred activity. Recruiting staff will contact (via telephone) the trial allocation office in charge of randomization once a participant has consented to participate and will be given the allocation. The allocation and sequence of allocation will be concealed from evaluators.

Evaluators who will be assessing the participants will be blinded to the hypotheses and to the group assignment of participants. Participants will be asked not to mention their group assignment to evaluators. Any breach of this group assignment blinding by participants will be noted, but since evaluators will be blinded from the hypotheses this should have minimal effect on integrity. Activity leaders will not be blinded to the study hypotheses. Although participants will know that they are in the Spanish, music, or documentary discussion group, they will not be informed of the study hypotheses, i.e., participants assigned to ENGAGE-DISCOVERY will be unaware that it is an active control condition. To keep participants blinded from the hypotheses and reduce the likelihood that the expectations of participants bias the data [29], close attention will be paid to ensure that the content of the intervention and the wording of recruitment documents and consent forms do not convey the expectations that one condition is inferior in terms of its effect on the brain and cognition.

### Intervention

The intervention sessions will take place at Baycrest in Toronto, at the IUGM in Montreal, or in community centers in both cities.

#### ENGAGE-MUSIC/SPANISH

ENGAGE-MUSIC/SPANISH is a 24-session program that combines leisure activities (music lessons or Spanish language lessons, and carefully selected videogames) and formal cognitive training. This intervention dose should be sufficient to show an effect based on prior studies using cognitive training similar to the one included here [10, 36, 37] or leisure-based intervention [38]. Each session will last approximately 2 h for a total of 48 training hours (see detailed schedule in Table 1). The content of the intervention was developed by a multidisciplinary team composed of neuroscientists, neuropsychologists, occupational therapists, speech therapists, and medical doctors specializing in gerontology and psychiatry. The training will be delivered in a group format of 5 to 9 participants. There will be 17 h devoted to cognitive training and 31 h to leisure activities. The program will be delivered over 16 weeks with two sessions per week during the first 8 weeks and one weekly session during the following 8 weeks. Training will be supplemented by homework (about 2 h per week). There are no criteria suggesting that the intervention should be discontinued or modified for a participant.

**Table 1:** The ENGAGE-MUSIC/SPANISH program: class and home sessions

Week	First class of the week		Homework	Second class of the week		Homework
	Hour 1	Hour 2		Hour 1	Hour 2	
1	**Educational-1: What are attention and memory and how they are affected by age?**		Formal (60 min)	**Educational-2: Lifestyle factors influencing cognition**		Formal (60 min)
2	**Attention-1: Priority strategy**	Videogames (priority strategy): Neuropeak	Formal (30 min) + Neuropeak (30 min)	**Attention-2: Priority strategy**	Videogames (priority strategy): Tap to Cook	Formal (30 min) + Tap to Cook (30 min)
3	**Attention-3: Priority strategy**	Videogames (priority strategy): Neuropeak + Tap to Cook	Formal (30 min) + Neuropeak (30 min)	**Attention-4: Priority strategy**	Videogames (priority strategy): Neuropeak + Tap to Cook	Formal (30 min) + Tap to Cook (30 min)
4	**Memory-1: Introduction of all strategies + see it and say it + habits + memory aids**		Formal (30 min) + Neuropeak (30 min)	Music or Spanish-1 (see it and say it)		Music/Spanish (30 min) + Tap to Cook (30 min)
5	**Memory-2: Spaced retrieval**	Music or Spanish-2 (spaced retrieval)	Formal (30 min) + Neuropeak (30 min)	Music or Spanish-3 (spaced retrieval)		Music/Spanish (30 min) + Tap to Cook (30 min)
6	**Memory-3: Make it meaningful and mental imagery**	Music or Spanish-4 (make it meaningful)	Formal (30 min) + Neuropeak (15 min) + Chicken Run (15 min)	Music or Spanish-5 (mental imagery)		Music/Spanish (30 min) + Tap to Cook (30 min)
7	**Memory-4: Face-name association**	Music or Spanish-6 (face-name association)	Formal (30 min) + Neuropeak (15 min) + Chicken Run (15 min)	Music or Spanish-7 (face-name association)		Music/Spanish (30 min) + Tap to Cook (30 min)
8	**Memory-5: Method of Loci**	Music or Spanish-8 (method of Loci)	Formal (30 min) + Neuropeak (15 min) + Chicken Run (15 min)	Music or Spanish-9 (method of Loci)		Music/Spanish (30 min) + Tap to Cook (30 min)
9	**Memory-6: Review all strategies**	Music or Spanish-10 (see it and say it)	Formal (30 min) + Music/Spanish (30 min) + Tap to Cook (30 min) + Neuropeak (15 min) + Chicken Run (15 min)			
10	Music or Spanish-11 (spaced retrieval)		Music/Spanish (60 min) + Tap to Cook (30 min) + Neuropeak (15 min) + Chicken Run (15 min)			
11	**Memory-7: Review/scenarios**	Music or Spanish-12 (make it meaningful)	Formal (30 min) + Music/Spanish (30 min) + Tap to Cook (30 min) + Neuropeak (15 min) + Chicken Run (15 min)			
12	Music or Spanish-13 (mental imagery)		Music/Spanish (60 min) + Tap to Cook (30 min) + Neuropeak (15 min) + Chicken Run (15 min)			
13	Music or Spanish-14 (face-name association)		Music/Spanish (60 min) + Tap to Cook (30 min) + Neuropeak (15 min) + Chicken Run (15 min)			
14	Music or Spanish-15 (method of Loci)		Music/Spanish (60 min) + Tap to Cook (30 min) + Neuropeak (15 min) + Chicken Run (15 min)			
15	Music or Spanish-16 (free use of strategy)		Music/Spanish (60 min) + Tap to Cook (30 min) + Neuropeak (15 min) + Chicken Run (15 min)			
16	**General review**	Music or Spanish-17 (free use of strategy)	Not applicable			

Bold typeface corresponds to formal cognitive training. Trained memory and attention strategies are in parentheses

##### Formal cognitive training

Cognitive training sessions will comprise 4 h of psychoeducation on cognitive aging, 4 h of attentional training, 8 h of memory training, and 1 h for general review. Psychoeducation sessions will include courses about the brain and how aging affects memory and attention, as well as tips for a healthier lifestyle to promote successful aging (sleep, diet, cognitive engagement, physical activity, social life, depression and stress management, etc. [12, 39]). Training for attention and memory will focus on strategies that were found to improve cognition in older adults. The attention training component relies on computerized exercises taken from Gagnon and Belleville [40] and Bherer et al. [41]. In these exercises, participants practice attentional control in dual-task situations by varying the amount of attention they allocate to one task over the other (priority strategy; e.g., allocate 80% of one’s attention to task A and only 20% to task B) in different blocks of practice. Participants will receive feedback on their performance to increase awareness of their ability to control their attention. The “memory” component of the program is taken from the MEMO program [10, 12] and from the Memory and Aging Program [39, 42]. Participants learn how to maximize their memory performance by using internal and external strategies. Internal strategies will include: learning how to visualize and/or formulate an intention out loud to maximize the chances of remembering it later (“see it and say it”); learning “spaced retrieval”, which is a strategy that consists of progressively increasing the delay between retrieval trials to strengthen encoding of new information; learning how to create semantic connections between new information and existing knowledge; and learning “mental imagery” by creating mental images that associate two elements that need to be memorized in tandem. Mental imagery will be more specifically used to remember a person’s face and name, and to remember lists (method of loci). External strategies will include the use of memory aids (e.g., agenda, to-do lists) and ways to organize the environment in a manner that facilitates memory (e.g., creating routines).

##### Leisure activities

**Fig. 2:**
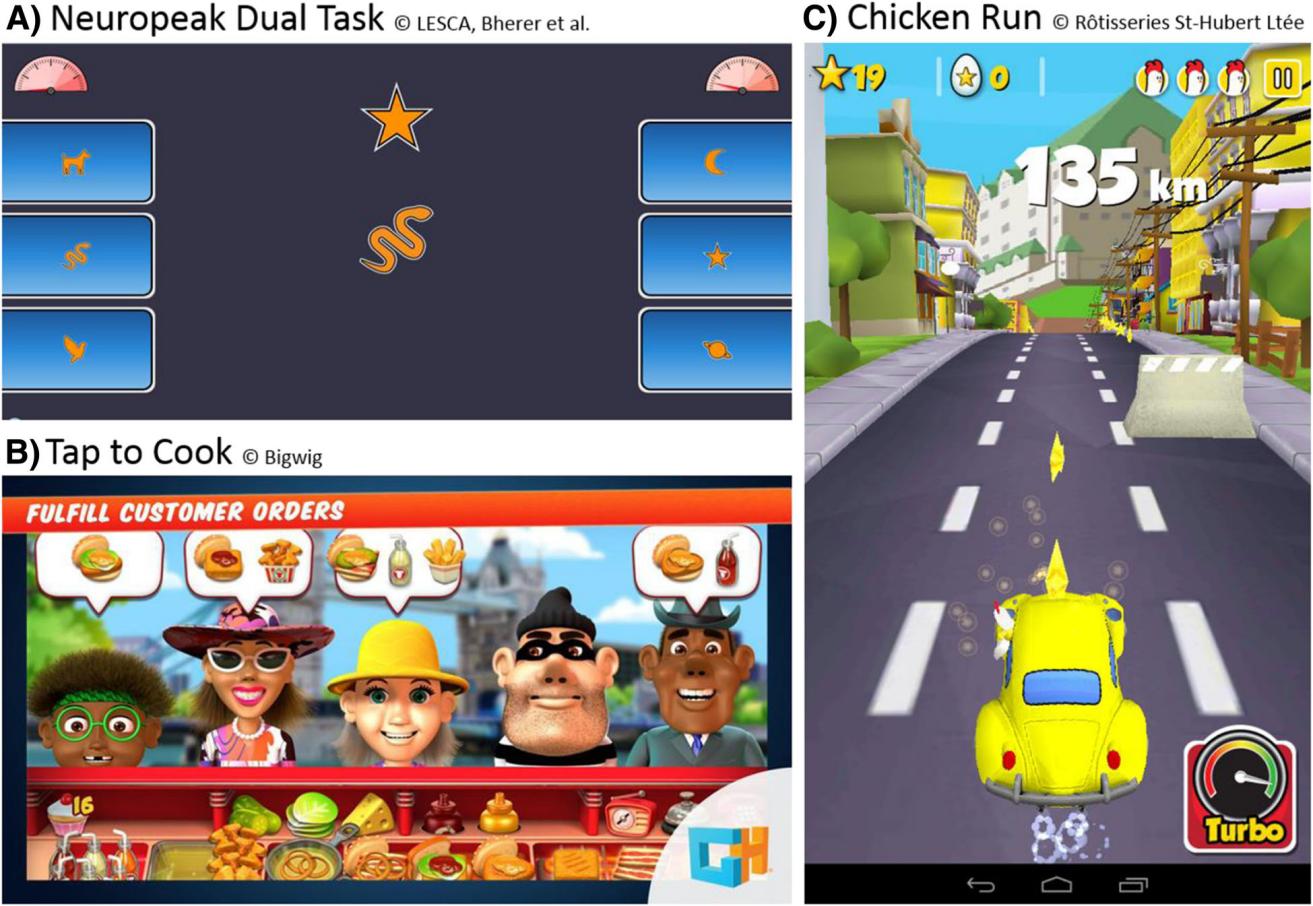
Illustration of the videogames used to train the ‘priority’ attentional strategy. **a** Neuropeak dual task; **b** “Tap to Cook”; **c** “Chicken Run”

The 31 h of leisure activity classes will comprise 4 h of casual videogames and 27 h of either music or Spanish training. Videogames are played on electronic tablets and include: (1) the Neuropeak dual task (Lussier M, Bherer L, et al: Normative data for a tablet-based executive functions assessment battery in healthy older adults, forthcoming), where the participant has to respond to images presented in the center of the screen by pressing the corresponding images on his/her left (with the left hand) or on his/her right (with the right hand); (2) Tap to Cook, which is a commercially available game where the player is in charge of a food truck and has to prepare and serve food to customers according to their orders; and (3) Chicken Run, a commercially available game where the player is driving a car on a road full of obstacles. These games have been selected because they are demanding in terms of working memory and/or processing speed, and because they have components of dual tasking, and thus will allow to train the ‘priority’ attentional strategy (for instance, prioritizing serving burgers over side orders in Tap to Cook, and then switch priority). See Fig. 2 for an illustration of each game interface. Tablets will be provided to the participants for the duration of the study.

Music and Spanish activities were selected because the literature on the neuroscience of aging indicated that they may have a neuroprotective effect. Music and Spanish sessions have been created by professional music and Spanish teachers to be ecologically valid, similar to a class that adult beginners would receive in the community. Music sessions include mostly practical exercises; for example, choral singing, introduction to musical composition and song writing, rhythmical exercises (e.g., introduction to drums, light dancing, body percussions) but also some more theoretical elements (for instance, introduction to music notation, visual representation of pitch and rhythm, musical styles, composers, etc.). The Spanish sessions have a similar proportion of practical and theoretical teaching. Most of the sessions contain practical exercises which include an introduction to simple grammatical notions and vocabulary, taught in ecologically valid situations involving simple conversations (e.g., sharing with the group about us, expressing in Spanish where we come from, what we like, what we want to do, etc.), or role playing (e.g., ordering food in a restaurant, asking for directions, etc.), as well as an introduction to some elements of the Hispanic culture worldwide (food, art, etc.).

Music or Spanish sessions alternate with cognitive training (see Table 1) so that the strategy learned during cognitive training can be subsequently applied during the sessions devoted to leisure. The attentional strategy will be applied while playing casual videogames as the three videogames have components of dual tasking. Hence, participants will be asked to prioritize the left or the right hand-side images in the Neuropeak dual task and certain actions over others in the Tap to Cook and Chicken Run games. Memory strategies will be applied in the music/Spanish sessions. For instance, mental imagery will be used to memorize song lyrics and to learn Spanish vocabulary, face-name association will be used to memorize faces of music composers and Hispanic figures, and spaced retrieval will be used to learn the time value of musical notes or irregular verbs in Spanish.

##### Homework

To increase the likelihood of transfer, participants will be asked to complete exercises meant to review and practice the learned strategies by applying them to everyday life situations (about 2 h of homework per week in total). For instance, they will be asked to use the face-name association technique to remember the name of a new person they encounter. Homework will also include music/Spanish exercises to consolidate the concepts learned during the class and to keep applying the memory strategies to the leisure activities. Finally, participants will be asked to practice the attention control strategy on the tablet games (1 h per week).

#### ENGAGE-DISCOVERY

The ENGAGE-DISCOVERY program (Table 2) will be used as an active control. This will be structured in the same manner as the ENGAGE-MUSIC/SPANISH program and will comprise the same number of classes, hours of homework, and ratio of formal classes to leisure activities. The content of the ENGAGE-DISCOVERY program was selected to be less cognitively stimulating than the intervention, yet interesting and enjoyable, to ensure the same level of expectation and participation.

**Table 2:** The ENGAGE-DISCOVERY program: class and home sessions

Week	First class of the week		Homework	Second class of the week		Homework
	Hour 1	Hour 2		Hour 1	Hour 2	
1	**Educational-1: Myths and reality about aging; discover your brain**		Formal (60 min)	**Educational-2: Attention and working memory**		Formal (60 min)
2	**Educational-3: Semantic memory**	Videogames: Freecell	Formal (30 min) + Freecell (30 min)	**Educational-4: Episodic memory**	Videogames: Words	Formal (30 min) + Words (30 min)
3	**Educational-5: Memory and senses**	Videogames: Freecell + Words	Formal (30 min) + Freecell (30 min)	**Educational-6: Procedural memory**	Videogames: Freecell + Words	Formal (30 min) + Words (30 min)
4	**Educational-7: Executive functions**		Formal (30 min) + Freecell (30 min)	Documentary and discussion		Reading (30 min) + Words (30 min)
5	**Educational-8: Neuroplasticity**	Documentary and discussion	Formal (30 min) + Freecell (30 min)	Documentary and discussion		Reading (30 min) + Words (30 min)
6	**Educational-9: Nutrition and exercise**	Documentary and discussion	Formal (30 min) + Freecell (15 min) + Equilibrium (15 min)	Documentary and discussion		Reading (30 min) + Words (30 min)
7	**Educational-10: Sleep and stress management**	Documentary and discussion	Formal (30 min) + Freecell (15 min) +Equilibrium (15 min)	Documentary and discussion		Reading (30 min) + Words (30 min)
8	**Educational-11: Health and mood**	Documentary and discussion	Formal (30 min) + Freecell (15 min) + Equilibrium (15 min)	Documentary and discussion		Reading (30 min) + Words (30 min)
9	**Educational-12: Review—different types of memory**	Documentary and discussion	Formal (30 min) + Reading (30 min) + Words (30 min) + (15 min) + Equilibrium (15 min)			
10	Documentary and discussion		Reading (60 min) +Words (30 min) + Freecell (15 min) + Equilibrium (15 min)			
11	**Educational-13: Review—attention and executive functions**	Documentary and discussion	Formal (30 min) + Reading (30 min) + Words (30 min) + Freecell (15 min) + Equilibrium (15 min)			
12	Documentary and discussion		Reading (60 min) + Words (30 min) + Freecell (15 min) + Equilibrium (15 min)			
13	Documentary and discussion		Reading (60 min) + Words (30 min) + Freecell (15 min) + Equilibrium (15 min)			
14	Documentary and discussion		Reading (60 min) + Words (30 min) + Freecell (15 min) + Equilibrium (15 min)			
15	Documentary and discussion		Reading (60 min) + Words (30 min) + Freecell (15 min) + Equilibrium (15 min)			
16	**General review**	General discussion	Not applicable			

Bold typeface corresponds to formal (educational) sessions

During the 17 h of formal classes, participants will receive information on the brain and cognitive processes, the effect of age on cognition, and tips to promote successful aging (diet, sleep, physical activity, etc.). The 31 h of leisure activities will comprise documentaries and discussion as well as low-stimulating casual videogame playing. Participants will be watching and discussing Planet Earth (a TV series produced by the BBC) and Tomorrow (produced by Moviemovie) documentaries which will cover various topics of interest about nature (geography, animals, landscapes, climate, environment). A discussion-debate will follow each documentary where participants will be invited to share their opinion on topics related to the documentary they have seen. This is to ensure that participants have a similar level of interaction as in the music and Spanish classes. In addition, participants will also play videogames on tablets: (1) FreeCell, where participants have to organize cards in series; (2) Words, where participants have to find words hidden in grids; and (3) Equilibrium, where they have to remove pieces from a structure while maintaining its balance. The games selected are entertaining but, unlike the games selected for the active intervention, do not include formal dual-tasking, processing speed, or metacognitive control. Homework will involve exercises related to the information contained in the psychoeducation sessions (e.g., making a sleep journal), as well as playing the three videogames.

### Adherence to treatment and retention

A number of strategies will be used to reduce attrition and promote retention over the 24-month follow-up. The coordinator of the study will be available by phone to discuss potential problems. In case of reduced adherence, we will contact the participant or his/her informant to identify the reasons and/or barriers that reduce their attendance and help identify solutions. The choice of a preference trial where participants are given the possibility to exclude an activity they dislike was also made to increase adherence to treatment. The team will remain in contact with the participants during the long-term follow-up through yearly telephone calls and greeting cards for the festive season. The attrition rate is expected to be approximately 18% based on our most recent study which used a similar program [10]. The characteristics of those who withdraw will be analyzed and intent-to-treat analyses will be used. Participants who withdraw from the program will be invited to come for follow-up assessments.

### Baseline characterization

Different measures will be used at baseline for diagnosis and clinical characterization and for measuring outcomes. Participants will be characterized on a number of psychological, cognitive, and physical variables, as well as past and current lifestyle and activities (please refer to ClinicalTrials.gov NCT03402919 and to Additional file 2: Appendix 1 for a description of the baseline assessments). This will include information on demographics (age, sex, education), occupations and hobbies, social engagement, general health, current and past medication, medical history, family medical history, physical measurements (weight, blood pressure, etc.), neurological examination, blood, saliva and urine collection (and an optional lumbar puncture), screening tests for mild cognitive impairment or dementia, gait evaluation, sensory processing (visual and auditory acuity), smoking and alcohol use, nutritional habits (Canadian Longitudinal Study on Aging (CLSA) Short Diet Questionnaire [43], and abbreviated version of the SCREEN II [44]), physical exercise (Physical Activity Scale for the Elderly (PASE) [45] and California Teachers Study Long Term Recreational Physical Activity Survey [46]), self-perception and psychological state (self-administered questionnaires, including Beck Depression Inventory II (BDI-II) [47] and Beck Anxiety Inventory (BAI) [48]). Biosamples will allow investigation of genetic biomarkers of a high risk of Alzheimer’s disease and of brain plasticity (apolipoprotein (Apo)E4, brain-derived neurotrophic factor (BDNF), and catechol-O-methyltransferase (COMT)). Cognitive reserve proxies will be evaluated using years of education and a modified version of Rami and colleagues cognitive reserve questionnaire adapted for French and English by the CIMA-Q team [32, 49]. As the intervention involves training participants on videogames and music and/or second language learning, the participants prior experience in those areas will be characterized through questionnaires of game literacy (Game Experience Questionnaire, created by our team), computer proficiency (Computer Proficiency Questionnaire (CPQ) [29]), experience with music (Music Experience Questionnaire, created by our team), and multilingualism (Language Experience and Proficiency Questionnaire (LEAP) [50]).

### Outcome variables

Evaluations will be conducted at the Centre for Memory and Aging, Baycrest Health Sciences, and Sunnybrook Hospital for Toronto participants. In Montreal, they will be conducted at the IUGM. Outcome measures will be tested at three time points: before the intervention (PRE; time frame within 12 weeks before intervention starts), just after the intervention at the 4-month follow-up (F4; time frame within 8 weeks after the end of the intervention), and at the 24 follow-up (F24; time frame 2 years after PRE ± 3 months). Participants are not allowed to participate in other research programs or lifestyle interventions between baseline and the first follow-up (F4), but they can get involved in any activities between F4 and F24.

#### Primary outcome measure

The primary outcome measure will be performance on a composite episodic memory score measured at PRE and F4. The composite score will be computed by averaging *z*-scores from the delayed recall of the Rey Auditory Verbal Learning Test (RAVLT) [51] and the delayed recall of the face-name association taken from the CIMA-Q study [32]. The RAVLT consists of learning a list of 15 unrelated words (list A) over five trials, followed by one-trial learning and recall of an interference list (list B). Free recall of list A is tested immediately after recalling list B, and again 20 min later for delayed recall. The score that will be converted into a *z*-score to compute the composite score is the number of words recalled in the delayed trial (maximum score = 15). In the face-name association task, participants learn nine pairs of face + first name associations. They are asked to recall the name associated with the faces and to recognize the pairs among distractors immediately after learning and following a 20-min delay. The score obtained which will be used in the composite score will be the number of names recalled in the delayed trial (maximum score = 9).

#### Secondary outcomes

##### Attentional control

Attentional control will be measured at PRE, F4, and F24 assessments. A composite score will be computed by averaging the *z*-scores obtained from the Number-letter task (switching cost) [52], the Complex (four-choice) reaction time (RT) from the CLSA Reaction Time Task [53], and an adapted Flanker task comprising a dual-task condition (congruency effect and dual task cost; from Doody AM, Rivest J, Leach L: Selective and divided attention, within and between the visual and auditory modalities, of individuals aging with mild cognitive impairment, unpublished). The Number-letter task assesses the participants ability to shift attention. Participants are presented with pairs made of one number and one letter (e.g., 8A) and must indicate whether the number is odd or even, or whether the letter is a consonant or a vowel, depending on the location of the pair on the screen. Shifting cost will be determined by subtracting RTs to non-shift trials (i.e., same processing as previous trial) from RTs to shift trials. The CLSA RT task measures one-choice and four-choice RT. The average RT on four-choice trials will be taken to reflect speed of processing for the attentional control composite score. In the adapted Flanker task, participants must indicate the direction of a central target arrow while ignoring a distractor arrow that is presented to the left or right of the target on one third of the trials. In the congruent condition, both arrows are pointing in the same direction, while in the incongruent condition, the distractor arrow points in the direction opposite to the target arrow. Inhibition cost is measured by subtracting RTs to congruent trials from RTs to incongruent ones. The divided attention task requires participants to complete the same Flanker task, while also monitoring drawings representing everyday objects and animals that are shown above the arrows. Participants are asked to monitor the drawings and to withdraw their response when a drawing of a dog is presented. Divided attention cost is obtained by subtracting RT to focused attention trials from RT to divided attention trials.

##### Psychological health

Four categories of psychological health measures will be taken at PRE, F4, and F24. (1) An anxiety and depression composite score will be calculated from *z*-scores obtained from the Geriatric Anxiety Inventory (GAI) [54] and the Geriatric Depression Scale (GDS) [55]. (2) A score of apathy will be computed by combining results from the participant and informant versions of the Apathy Inventory [56]. (3) Quality of life will be obtained by computing a combined score using the Quality of Life Alzheimer Disease questionnaire (QoL-AD) [57] and the 36-item short-form health survey (SF-36) [58]. (4) Finally, help-seeking behavior will be assessed by the Medical Care section of the Stanford Chronic Disease questionnaire [59].

##### Impact and transfer to everyday life

The impact of the intervention on the everyday life of participants will be measured at PRE, F4, and F24, with both self-administered questionnaires and performance-based tasks. Seven scores will be computed. (1) Self-administered questionnaires on memory functioning will include the Metamemory Questionnaire (MMQ) [60] and will be used to assess the perceptions of participants about their memory, common memory mistakes, and their use of memory strategies. (2) An abbreviated version of the Auto-administered Memory Questionnaire (AMQ) [61, 62] will assess cognitive (memory and attention) complaints. (3) A questionnaire measuring complex activities of daily living, the Alzheimer’s Disease Cooperative Study—Activities of daily living—Prevention Instrument (ADCS-ADL-PI) [63], will be completed by the participants and their informant and will assess the ease with which the participants perform instrumental activities of daily living. A composite score will be calculated for this test by combining both participant and informant scores. (4) Participants will complete the Cognitive Activities Questionnaire, a questionnaire about the amount of cognitive activities performed in everyday life, adapted from Vemuri and colleagues [64]. (5) Jessen’s questions [20] will be asked. The measure that will be used is the number of participants that have a memory concern (i.e., answering “yes” to both of the Jessen questions [20, 21]) at each time point. Performance-based measures will include two measures. (6) In the Memory Toolbox test [39], participants are presented with everyday scenarios commonly encountered and asked to generate memory strategies that would be beneficial. Participants are scored based on the number and appropriateness of the memory strategies generated. (7) The Direct Assessment of Functional Status—Revised (DAFS-R) [65] is a test of instrumental activities of daily living assessing skills in four domains: communication, financial management, shopping, and medication management. The test simulates everyday life activities, such as looking for a telephone number in a phone book and calling this number from an unplugged telephone, preparing a mock cheque or balancing a mock bank account, memorizing a list of things to buy and retrieving these items on a shelf containing empty boxes of food (targets and distractors), preparing a pill box, etc.

##### Brain structure

Hippocampal volume and cortical thickness will be measured at PRE, F4 and F24 for a subgroup of participants (*N* = 54; 27 per intervention condition) at the Montreal site. Participants will be scanned on a Siemens TIM Trio 3-T PRISMA MRI system (Siemens Medical Solutions, Erlangen, Germany) at the Unité de Neuroimagerie Fonctionnelle (UNF) of the IUGM Research center. A structural sequence will be used to measure hippocampal volume (mm^3^) and cortical thickness (mm) since these are early biomarkers of AD [66] and were found to be sensitive to memory training (see, for example, [67]).

##### Brain function

A task-related fMRI activation sequence will be administered at PRE and F4 for a subgroup of participants (*N* = 54). This will measure activation while participants perform an associative memory encoding task. In this task, which was developed by CIMA-Q [32], participants are presented with a random sequence of 78 images and control stimuli (grey squares) for 3 s each. Each image appears within one of the four quadrants of the screen and participants are asked to remember the image and its position. Retrieval is tested outside the scanner after a 10-min delay where participants see series of 80 targets and 20 foils and are asked to indicate whether the image was presented and its position. An event-related model will allow analysis of activation as a function of whether the item and its position were correctly recognized (i.e., “correct item/correct position” versus “correct item/wrong position” and “incorrect item”).

##### Expectation measure

We will assess the expectations of participants regarding the training program to which they participated using a questionnaire adapted from prior studies [29, 68] administered at PRE and F4. The questionnaire consists of 10 questions using 0–6 answer scales. The expectation score (max = 60) will be compared between groups.

### Data storage and data transfer procedure

All the data collected will be included in the CCNA Longitudinal Online Research and Imaging System (LORIS) system [69] which will house the data from every COMPASS-ND participating institution. It will allow direct data entry from the two study sites into a single centralized system accessible by both teams. LORIS is a web-based database solution for neuroimaging and other research data that is physically located at McGill University in Montreal. Information and datasets are stored in the LORIS database under strict security provisions. Safeguards are in place to minimize the risk of a breach in the security of this database system resulting in the access of information. In the unlikely case of a security failure, research participants would be immediately notified by the site investigator. This procedure is described in the informed consent signed by the participants at study entry.

To ensure quality of data entry and error corrections, a double-entry procedure will be used. This procedure requires two different persons to enter the same set of data from the paper or computer files used during assessment. Any inconsistency is flagged by the system and the conflict will be resolved by confirming the correct data is entered into the system.

### Safety procedures

All data will be de-identified. Study participants will be assigned a unique anonymized study identification number that will be used to store their data. Brain images will be processed to remove any direct identifiers of an individual study participant. Information about study participants will be made publicly available to the extent permitted by the applicable laws and regulations. In CCNA publications, only group data will be reported. In cases where participant identification is required (e.g., when an incidental finding is uncovered on coded data), the recruiting site study investigator will be informed and be given access to the minimum identifying information required to associate the incidental finding to the participant identity and move it toward a resolution. For data stored locally at the training site, all documents will be anonymized and stored in a locked filing cabinet in a locked room, or on an encrypted and password protected computer, with access strictly restricted to the research team coordinators. Electronic files which contain names and contact information of participants will be encrypted and password protected and stored in a password protected desktop computer at the training site (IUGM research center and Baycrest). No identifying personal information will be disclosed in any resulting publication or presentation.

With the participants’ written consent, audio- or video-recordings will be collected during the evaluation or training sessions for quality control or training purposes. These files will be kept on an encrypted and password protected computer under the responsibility of the principal investigator, with access strictly restricted to the research team coordinators. These files will be kept for analysis until the end of the project and then will be deleted from the computer. No transcription will be done. With the participant’s consent, some of the files recorded during the intervention sessions could be used for teaching, research, or scientific conferences. In this case, these specific files will be kept for 25 years.

### Quality control and monitoring

The programs are manualized to ensure consistency between sites and between trainers. Activity leaders for the cognitive sessions of ENGAGE-MUSIC/SPANISH and for the ENGAGE-DISCOVERY program will have a professional background in occupational therapy and/or psychology and neuropsychology. They will receive 20 h of training and will be asked to complete mock trials of the training sessions. Two people will lead the programs (one person in each city), except for the music and Spanish activities that will be led by professional music and Spanish teachers since they require specific expertise. The music and Spanish teachers will receive a 10-h training program covering the leisure manuals and the memory strategies that will be used in their course. All intervention sessions will be videotaped or audio-recorded for quality control purposes. Monitoring will be done on random sections (covering 20% of the program) to ensure adherence to manuals.

Assessors who will collect data for clinical measures, outcomes, and moderating variables will be trained by the COMPASS-ND team and by the research coordinator of the research team. For COMPASS-ND testing, they will be required to obtain COMPASS-ND certification. This certification includes 40 h web-training (involving reading about the tests, watching demo videos, answering quizzes, and practicing task scoring from the demo videos), completion of a mock testing session which will be videotaped, and live observation (though a webcam) during the first evaluation for assessment of adherence to protocol. All testing sessions with participants will be audio-recorded and random portions will be monitored for protocol compliance.

The quality control for recruitment and evaluation is done by a senior research coordinator. The quality control for intervention integrity is ensured by two clinicians (AM and BG) with expertise in the content of the interventions.

Any potential change in the protocol (current version V2, December 2018) will be handled by the central coordinator who will be in charge of ensuring that the information is transmitted to the two sites and reported to the ethics committee in the form of an ethics amendment request.

The CCNA COMPASS-ND study is audited yearly by an independent external scientific review committee.

### Statistical analyses

#### Sample size

The trial will recruit 144 participants. The sample size was estimated from a power analysis with G*power based on pilot findings collected with the MEMO program [10, 12], using word recall and face-name associations tasks. The MEMO program is included as part of the formal training in this intervention in addition to stimulating leisure activities and attentional training. Therefore, it is reasonable to expect that the effect should be at least as large as the one observed when using MEMO alone. The pilot data indicated a medium effect size for the group-by-time interaction when examining face-name association and a large effect size when examining word recall (see Additional file 2: Appendix 2). It shows that the sample size would be sufficient to detect an interaction given a similar effect size considering the same attrition rate that we obtained in this prior study (18%). Half of the sample will be recruited in Montreal and the other half will be recruited in Toronto.

#### Analysis of efficacy and maintenance on primary and secondary outcomes

Descriptive statistics will be used for demographics and baseline characteristics, with means and standard deviations given. We will compare the intervention groups on these variables using one-way analysis of variance (ANOVA) for continuous variables and Chi-square analyses for discrete variables. The primary efficacy analysis will be done with a modified intention-to-treat approach retaining all participants and identifying the characteristics of participants withdrawing from follow-up as well as the causes for withdrawal. As proposed for preference trials, a first set of analyses will only use fully randomized participants. This will be followed by analyses of the comprehensive cohort which will include those in the preference arms.

The linear mixed model will be used to assess the efficacy of the intervention. The mixed linear model has many advantages over typical ANOVA; it handles correlated data and is robust to unbalanced design, hence allowing for the inclusion of all participants, including those missing at follow-up. Missing data will not be imputed as mixed models use all available data, including those from participants having incomplete follow-up. The fixed effects will be intervention (ENGAGE SPANISH/MUSIC versus ENGAGE DISCOVERY), time (PRE versus F4), and their interaction. A significant interaction is expected if the intervention is more beneficial than the control condition. When an interaction is found, we will look at whether there is a significant difference between PRE and F4 in each group and assess group differences on change scores at post-training ([PRE – F4]/|PRE|). Efficacy is supported if the F4 change score is larger in the intervention group than in the control group. All analyses will be adjusted for site and baseline performance.

A similar approach will be taken for most of our secondary objectives. A set of secondary objectives of the study is to assess the long-term maintenance of the effect of the training. This will use a mixed linear model to test long-term maintenance of intervention effects (PRE versus F24) on primary outcome, as well as separate models (with three levels of time: PRE, F4, and F24) on measures of attentional control, psychological health variables (anxiety/depression, apathy, quality of life, help seeking), everyday functioning measures (MMQ, AMQ, ADCS-ADL-PI, the Cognitive Activities Questionnaire, Memory Toolbox, and DAFS-R), and on the structural brain measures (hippocampal volume and cortical thickness). Because the Jessen questions about subjective complaint are dichotomous, Chi-square analyses will be used to compare the number of participants who have a memory complaint before and after the intervention as a function of condition. For exploratory purposes and to inform future interventions, the above analyses will be repeated comparing the music and Spanish arms. The *p* values will be presented before and after adjustment for multiple comparisons based on the Hochberg procedure. All statistical tests will be two-tailed and a *p* value of less than 0.05 will indicate statistical significance. All computations will be made with SPSS version 23.0 (SPSS Inc., Chicago, IL, USA).

Functional MRI data will be subjected to standard pre-processing steps using SPM8 (i.e., motion correction, slice timing correction, co-registration with anatomical T1 image, spatial normalization). First level (individual) analyses will involve general linear models with box-car responses, convolving the time course of the event-related response per condition with the canonical hemodynamic response function. Second level (group) analyses will treat participants as a random variable, with each contrast tested at *p* < 0.05 after whole-brain, family-wise error correction. A cluster threshold of 50 or more contiguous voxels will be applied.

Preliminary analyses of the descriptive characteristics and baseline performance will be done after full recruitment is completed. Analysis of efficacy will be done at the end of the 4-month follow-up and long-term analyses will be done at the end of the 24-month follow-up.

#### Analysis of moderators

A small set of time-invariant variables will be included as predictors to assess their effects on primary and secondary outcomes. Three pre-specified subgroup analyses will be done according to sex (male versus female), education (less versus more than 12 years), and age (younger versus older than 75 years at baseline). Other subgroup analyses will be exploratory, such as reserve score based on the adapted version of the Rami and colleagues cognitive reserve questionnaire [32, 49], genetic markers of brain plasticity and risks for dementia (APOe4, BDNF, and COMT), baseline brain structure and function, depression (BDI-II [47]), and anxiety (BAI [48]). We will also include the expectations of participants regarding the cognitive training, compliance with the intervention (percentage of sessions attended and percentage of homework completed), amount of knowledge acquired during the intervention, and self-efficacy perception (personality measures). Stepwise regression analyses with the per-protocol participants from the cognitive training group will be used to explore whether the moderators predict delayed memory change scores at the two follow-up time points.

### Dissemination of study data

Data will be presented in international conferences and through publications in journals with peer-reviewed committees (see dissemination plan in Additional file 2: Appendix 3). Study results will also be presented to the public through lay-audience talks and press releases.

## Discussion

The ENGAGE study is a randomized, controlled, double-blind comprehensive cohort design trial to assess the efficacy and the long-term effect of a leisure-based training program in older adults with a memory complaint. The program is multifaceted and combines stimulating leisure activities with strategy-based cognitive training focusing on memory and attention. The overarching objective of the study is to provide empirical support that participation in cognitively engaging activities later in life can promote cognitive health, reduce cognitive decline, and build cognitive reserve.

While there have been a few studies that have shown the efficacy of cognitive interventions for persons with MCI, there has been a limited number of studies that have designed interventions for individuals with SCD or early MCI. However, SCD and early MCI have been suggested to represent a very early stage of future cognitive decline in some individuals, which also means that it is a period where the brain may have its greatest potential for plasticity effects. This makes SCD and early MCI a key phase for cognitive engagement and learning of strategies for memory and attention optimization.

This study has many strengths. One of them is the inclusion of an active control condition and strategies used to reduce and control for the expectation effects of participants. The majority of intervention studies use a no-contact or wait-list control group comparison, a design that does not allow control for the effects of social stimulation, expectations, or additional stimulation from everyday routine. By including an active control condition that mirrors the structure and the level of social engagement of the intervention condition, while providing the same expectations in terms of potential benefits, this study is in a better position to confidently attribute any positive results to the content of the program. This study will also measure generalization of cognitive benefits to everyday tasks, which is an important indication of the impact of the intervention. Furthermore, the study includes a number of approaches to increase transfer to activities of daily living, for instance practicing the cognitive strategies during the leisure sessions and including homework for practice in the home environment of participants. It will also measure the neural substrates of training-related benefits by using MRI and fMRI, which will contribute to building knowledge on the brain mechanisms by which such interventions exert their effects. Finally, few studies have assessed the long-term effect of cognitive interventions up to more than a year after training and, thus, the 24-month assessment included here will provide critical information on the maintenance of the effects as well as their impact on cognitive decline.

An important component of this study is to enroll participants with limited education to compare training efficacy as a function of cognitive reserve proxies. This is critical because cognitively stimulating interventions might be particularly beneficial to those individuals who are at greater risk of developing dementia. Furthermore, results will be more representative of the general population and hence more generalizable. Finally, as the population will be well characterized, it will be possible to assess whether some genetic, cognitive, or demographic characteristics are associated with a better response to treatment. This will allow provision of recommendations for the type of older adult who is most likely to benefit from such intervention.

In spite of these strengths, the study has some potential limitations. Although it offers several leisure activities, and the possibility of excluding one of them should increase motivation and engagement of participants, there is still the possibility that they will find neither music nor Spanish training appealing. This may potentially affect their engagement throughout the program and reduce generalization to real life, where people choose activities they enjoy from among a much larger number of options. Secondly, the training is relatively long, the inclusion of homework will increase burden, and the schedule is demanding. This might have an effect on recruitment success and on attrition rate. Sample size estimation was based on attrition from prior studies but those were different, and it is possible that attrition will be different in the present case. Third, although leisure activities were included to make the intervention appealing, cognitive training sessions may be tedious and increase withdrawal. Finally, while proficiency in Spanish is an exclusion criterion, it was not feasible to have bilingualism as an exclusion criterion because Canada is a bilingual country and Toronto and Montreal have culturally diverse populations. This is of some concern because there is substantial evidence that bilingualism may have a protective effect against dementia (e.g., [15, 70]) and thus bilingualism has the potential to be a confounding variable. However, it will be possible to use bilingualism as a confounding and moderating factor in the analyses if it is found to differ among groups and/or correlate with efficacy.

In conclusion, the purpose of the ENGAGE study is to provide evidence for the short-term efficacy and long-term maintenance of a leisure-based cognitive intervention. Additionally, ENGAGE will provide data regarding the impact of cognitively engaging activities on brain structure and function, as well as providing data on what characterizes good responders to intervention. The ENGAGE study targets individuals with a memory complaint who are at risk of developing AD. Positive results from this study will have major implications for the well-being and health of these individuals by encouraging them to participate in meaningful activities and by providing models for designing impactful interventions that can be used broadly in the community.

## Trial status

Protocol version number V2, December 2018. Recruitment began in September 2017. Date of recruitment completion is anticipated to be March 2020.

## Additional files

Additional file 1: SPIRIT 2013 checklist: recommended items to address in a clinical trial protocol and related documents. (DOC 122 kb)

Additional file 2: **Appendix 1.** COMPASS-ND procedures by visit. **Appendix 2.** Sample size. **Appendix 3.** Dissemination plan. **Appendix 4.** Consent form of the ENGAGE study (participant version). **Appendix 5.** Consent form of the ENGAGE study (informant version). (DOCX 58 kb)

## References

[1] Barnes DE, Yaffe K (2011). The projected effect of risk factor reduction on Alzheimer’s disease prevalence. Lancet Neurol.

[2] Stern Y (2003). The concept of cognitive reserve: a catalyst for research. J Clin Exp Neuropsychol.

[3] Stern Y (2009). Cognitive reserve. Neuropsychologia.

[4] Verghese J (2003). Leisure activities and the risk of dementia in the elderly. N Engl J Med.

[5] Wilson RS (2002). Participation in cognitively stimulating activities and risk of incident Alzheimer disease. Jama.

[6] Ball K (2002). Effects of cognitive training interventions with older adults: a randomized controlled trial. J Am Med Assoc.

[7] Willis SL (2006). Long-term effects of cognitive training on everyday functional outcomes in older adults. J Am Med Assoc.

[8] Plassman BL (2010). Systematic review: factors associated with risk for and possible prevention of cognitive decline in later life. Ann Intern Med.

[9] Simon SS, Yokomizo JE, Bottino CM (2012). Cognitive intervention in amnestic mild cognitive impairment: a systematic review. Neurosci Biobehav Rev.

[10] Belleville S (2018). MEMO+: efficacy, durability and effect of cognitive training and psychosocial intervention in individuals with mild cognitive impairment. J Am Geriatr Soc.

[11] Rebok GW (2014). Ten-year effects of the advanced cognitive training for independent and vital elderly cognitive training trial on cognition and everyday functioning in older adults. J Am Geriatr Soc.

[12] Belleville S (2006). Improvement of episodic memory in persons with mild cognitive impairment and healthy older adults: evidence from a cognitive intervention program. Dement Geriatr Cogn Disord.

[13] Carlson MC (2008). Exploring the effects of an “everyday” activity program on executive function and memory in older adults: Experience Corps®. The Gerontologist.

[14] Park DC (2014). The impact of sustained engagement on cognitive function in older adults: the synapse project. Psychol Sci.

[15] Craik FI, Bialystok E, Freedman M (2010). Delaying the onset of Alzheimer disease: bilingualism as a form of cognitive reserve. Neurology.

[16] Amer T (2013). Do older professional musicians have cognitive advantages?. PLoS One.

[17] Hanna-Pladdy B, Gajewski B (2012). Recent and past musical activity predicts cognitive aging variability: direct comparison with general lifestyle activities. Front Hum Neurosci.

[18] Moussard A (2016). Life-long music practice and executive control in older adults: an event-related potential study. Brain Res.

[19] Anguera JA (2013). Video game training enhances cognitive control in older adults. Nature.

[20] Jessen F (2014). A conceptual framework for research on subjective cognitive decline in preclinical Alzheimer’s disease. Alzheimers Dement.

[21] Jessen F (2014). AD dementia risk in late MCI, in early MCI, and in subjective memory impairment. Alzheimers Dement.

[22] Huijbers W (2015). Amyloid-β deposition in mild cognitive impairment is associated with increased hippocampal activity, atrophy and clinical progression. Brain.

[23] O’brien J (2010). Longitudinal fMRI in elderly reveals loss of hippocampal activation with clinical decline. Neurology.

[24] Chandler M (2016). Everyday impact of cognitive interventions in mild cognitive impairment: a systematic review and meta-analysis. Neuropsychol Rev.

[25] Simons DJ (2016). Do “brain-training” programs work?. Psychol Sci Public Interest.

[26] Willis SL, Belleville S. Cognitive training in later adulthood. In: Handbook of the Psychology of Aging (Eighth Edition). San Diego: Elsevier. 2016. p. 219–43.

[27] Belleville S (2011). Training-related brain plasticity in subjects at risk of developing Alzheimer’s disease. Brain.

[28] Engvig A (2012). Memory training impacts short-term changes in aging white matter: a longitudinal diffusion tensor imaging study. Hum Brain Mapp.

[29] Boot WR (2013). The pervasive problem with placebos in psychology: why active control groups are not sufficient to rule out placebo effects. Perspect Psychol Sci.

[30] King M (2005). Impact of participant and physician intervention preferences on randomized trials: a systematic review. JAMA.

[31] Craig P (2008). Developing and evaluating complex interventions: the new Medical Research Council guidance. BMJ.

[32] Belleville S (2014). Detecting early preclinical Alzheimer’s disease via cognition, neuropsychiatry, and neuroimaging: qualitative review and recommendations for testing. J Alzheimers Dis.

[33] Elwood RW (1991). The Wechsler Memory Scale—Revised: psychometric characteristics and clinical application. Neuropsychol Rev.

[34] Chapman KR (2016). Mini mental state examination and logical memory scores for entry into Alzheimer’s disease trials. Alzheimers Res Ther.

[35] Petersen RC (2001). Current concepts in mild cognitive impairment. Arch Neurol.

[36] Lampit A, Hallock H, Valenzuela M (2014). Computerized cognitive training in cognitively healthy older adults: a systematic review and meta-analysis of effect modifiers. PLoS Med.

[37] Ball KK (2013). Speed of processing training in the ACTIVE study: how much is needed and who benefits?. J Aging Health.

[38] McDonough IM (2015). The synapse project: engagement in mentally challenging activities enhances neural efficiency. Restor Neurol Neurosci.

[39] Troyer AK (2001). Improving memory knowledge, satisfaction, and functioning via an education and intervention program for older adults. Aging Neuropsychol Cognit.

[40] Gagnon LG, Belleville S (2012). Training of attentional control in mild cognitive impairment with executive deficits: results from a doubleblind randomised controlled study. Neuropsychological rehabilitation.

[41] Bherer L (2005). Training effects on dual-task performance: are there age-related differences in plasticity of attentional control?. Psychol Aging.

[42] Wiegand MA (2013). Facilitating change in health-related behaviours and intentions: a randomized controlled trial of a multidimensional memory program for older adults. Aging Ment Health.

[43] Shatenstein B (2005). Development and validation of a food frequency questionnaire. Can J Diet Pract Res.

[44] Keller H, Goy R, Kane S (2005). Validity and reliability of SCREEN II (Seniors in the community: risk evaluation for eating and nutrition, Version II). Eur J Clin Nutr.

[45] Washburn RA (1999). The physical activity scale for the elderly (PASE): evidence for validity. J Clin Epidemiol.

[46] Dallal CM (2007). Long-term recreational physical activity and risk of invasive and in situ breast cancer: the California teachers study. Arch Intern Med.

[47] Beck AT, et al. Cognitive therapy of depression. New York: Guilford press; 1979. 10.1046/j.1440-1614.2002.t01-5-01015.x 11982561

[48] Beck AT (1988). An inventory for measuring clinical anxiety: the Beck Anxiety Inventory. J Consult Clin Psychol.

[49] Rami L (2011). Cognitive reserve questionnaire. Scores obtained in a healthy elderly population and in one with Alzheimer’s disease. Rev Neurol.

[50] Marian V, Blumenfeld HK, Kaushanskaya M (2007). The Language Experience and Proficiency Questionnaire (LEAP-Q): assessing language profiles in bilinguals and multilinguals. J Speech Lang Hear Res.

[51] Schmidt M (1996). Rey auditory verbal learning test: a handbook.

[52] Sylvain-Roy S, Lungu O, Belleville S (2014). Normal aging of the attentional control functions that underlie working memory. J Gerontol B Psychol Sci Soc Sci.

[53] Burton CL (2006). Intraindividual variability as a marker of neurological dysfunction: a comparison of Alzheimer’s disease and Parkinson’s disease. J Clin Exp Neuropsychol.

[54] Pachana NA (2007). Development and validation of the Geriatric Anxiety Inventory. Int Psychogeriatr.

[55] Yesavage JA (1983). Development and validation of a geriatric depression screening scale: a preliminary report. J Psychiatr Res.

[56] Robert P (2002). The apathy inventory: assessment of apathy and awareness in Alzheimer’s disease, Parkinson’s disease and mild cognitive impairment. Int J Geriatr Psychiatry.

[57] Logsdon RG (2002). Assessing quality of life in older adults with cognitive impairment. Psychosom Med.

[58] Ware JE Jr, Sherbourne CD. The MOS 36-item short-form health survey (SF-36): I. Conceptual framework and item selection. Med Care. 1992;30(6):473–83. 1593914

[59] Lorig K, et al. Outcome measures for health education and other health care interventions. United States: Sage; 1996.

[60] Troyer AK, Rich JB (2002). Psychometric properties of a new metamemory questionnaire for older adults. J Gerontol B Psychol Sci Soc Sci.

[61] Van der Linden M, et al. Un questionnaire d’auto-évaluation de la mémoire (QAM). 1989, Bruxelles: Editest.

[62] Clement F, Belleville S, Gauthier S (2008). Cognitive complaint in mild cognitive impairment and Alzheimer’s disease. J Int Neuropsychol Soc.

[63] Galasko D (2006). ADCS Prevention Instrument Project: assessment of instrumental activities of daily living for community-dwelling elderly individuals in dementia prevention clinical trials. Alzheimer Dis Assoc Disord.

[64] Vemuri P (2014). Association of lifetime intellectual enrichment with cognitive decline in the older population. JAMA Neurol.

[65] McDougall GJ (2009). The revised direct assessment of functional status for independent older adults. Gerontologist.

[66] Jack CR (1999). Prediction of AD with MRI-based hippocampal volume in mild cognitive impairment. Neurology.

[67] Engvig A (2010). Effects of memory training on cortical thickness in the elderly. Neuroimage.

[68] Rabipour S, Davidson PS (2015). Do you believe in brain training? A questionnaire about expectations of computerised cognitive training. Behav Brain Res.

[69] Das S (2012). LORIS: a web-based data management system for multi-center studies. Front Neuroinformatics.

[70] Klimova B, Valis M, Kuca K (2017). Bilingualism as a strategy to delay the onset of Alzheimer’s disease. Clin Interv Aging.

