# Supplementary material for: Rationale and protocol of the ENGAGE study: a double-blind randomized controlled preference trial using a comprehensive cohort design to measure the effect of a cognitive and leisure-based intervention in older adults with a memory complaint
Source: Trials. 2019 May 22;20:282. doi: 10.1186/s13063-019-3250-6 (PMC6532200; doi:10.1186/s13063-019-3250-6)
Supplement: Supplementary file 1 — SPIRIT 2013 checklist: recommended items to address in a clinical trial protocol and related documents. (DOC 122 kb) [file 13063_2019_3250_MOESM1_ESM.doc]

**APPENDIX 1: COMPASS-ND procedures by visit.**

* = Common measures between COMPASS and ENGAGE; ENGAGE-specific neuropsychological assessment will be done during an separate visit between COMPASS-ND visits 3 and 4.

**Visit 1: Screening and demographics (2-2.5 hours)**

- *Written informed consent prior to study procedures (study investigator or delegate of investigator; participant and study partner)
- *Assess inclusion/exclusion criteria (site staff; participant and study partner)
- *Audiometry (site staff; participant)
- *MoCA (all groups. SCI must have a score ≥ 25; all participants must have a score >13) (site staff; participant)
- *Subjective Memory Assessment (SCI only) (site staff; participant)
- *Logical Memory 1 & 2 from Wechsler memory scale (for SCI, MCI, V-MCI, AD, Mixed) (psychometrician; participant)
- *Sociodemographic Data
- Benson Figure Recall (for FTD, PD) (psychometrician; participant)
- *Physical activity questionnaires
- *CERAD word list Recall (for SCI, MCI, V-MCI, AD, Mixed) (psychometrician; participant)
- *Lawton Brody IADL scale (for SCI, MCI, & Vascular MCI) (site staff; study partner)
- *GDS 30
- *GAD 7
- *Clinical Dementia Rating scale (for SCI, MCI, V-MCI) (site staff; study partner and participant)
- Clinical PPA and bvFTD features from NACC Uniform Data Set FTLD Module (FTD) (physician or nurse; study partner)
- *Research Diagnosis
- *Provide participant with take-home assessment packet:
  - Hobbies and leisure activities (participant or study partner if the local investigator or staff determines that the participant is unable to complete the questionnaire)
  - Tobacco and alcohol consumption (participant or study partner if the local investigator or staff determines that the participant is unable to complete the questionnaire)
  - Activities of Daily Living (study partner)
  - Quality of Life (participant)
  - Oral Health (participant or study partner if the local investigator or staff determines that the participant is unable to complete the questionnaire)
  - End of Life Care (participant or study partner if the local investigator or staff determines that the participant is unable to complete the questionnaire)
  - Social network, support, & activities (participant or study partner if the local investigator or staff determines that the participant is unable to complete the questionnaire)
  - Adverse childhood experiences questionnaire (optional) (participant or study partner if the local investigator or staff determines that the participant is unable to complete the questionnaire)
  - Neuropsychiatric Inventory - Questionnaire (study partner)
  - Mild Behavioral Impairment Checklist (study partner)
  - Delirium questionnaire

**Visit 2: Clinical and Physical Assessments (2.5-3.5 hrs.)**

- *Fasting blood collection (nurse; participant)
- *Saliva collection (nurse; participant)
- *Urine collection (nurse; participant)
- *Physical Measurements (nurse; participant)
- *Health perception, fatigue, falls history & balance assessment (site staff; participant)
- *Buccal swab (optional)
- *Walking speed (4 and 6m) (site staff; participant)
- *Grip strength (site staff; participant)
- *Vision assessment (site staff; participant)
- *Hearing questionnaire & computer task (site staff; participant)
- *Olfaction assessment (site staff; participant)
- *Sleep (site staff; participant or study partner)
- *Cognitive fluctuations (site staff; study partner)
- *Nutrition (site staff, participant)
- Caregiver burden assessment (coordinator; participant [if in a caregiving role])
- *Current and past medications (site staff; participant or study partner)
- *Medical, mental health, and surgical history (site staff; participant or study partner)
- *Family history (site staff; participant or study partner)
- Initial Disease Symptoms (site staff; participant or study partner)
- Disease course (site staff; participant or study partner)
- Signs and Symptoms (physician; participant)
- *Physical examination (physician; participant)
- *Neurological examination (physician; participant)
- *Hachinski Ischemic Scale (physician; participant)
- *Clinical Diagnosis (physician)

**Visit 3: Neuropsychological Assessment**

- *WAIS-III Vocabulary Test
- *Rey Auditory Verbal Learning Test
- *Envelope Test
- *Trail Making Test A & B
- *Birmingham Object Recognition Battery Object Decision Task – Easy B
- *D-KEFS Color-Word Interference Test
- *Face-Name Association Task
- *WAIS-III Digit Symbol – Coding & Incidental Learning
- *Sentence Inhibition (Haylings) Task (computer test)
- *D-KEFS Category Fluency Test
- *D-KEFS Letter Fluency Test
- *Brief Visuospatial Memory Test – Revised
- *WAIS-III Digit Span Test
- *CCNA Reaction Time Task (computer test)
- *Social Norms Questionnaire
- *Judgment of Line Orientation Test
- *Noun and Verb Naming Subtests
- *Semantic Word-Picture Matching Test
- *Semantic Associates Test
- *Northwestern Anagram Test (Short Form)
- *Sentence Repetition Test
- *Word Reading Test
- *Boston Diagnostic Aphasia Exam Cookie Theft Picture Description
- *Sentence Reading Test
- Social Behavior Observer Checklist [FTD only]

**Visit 4: MRI scan**

- *T1
- *PD/T2
- *FLAIR
- *T2*
- *DTI (30 dir.)
- *BOLD Resting state

**Visit 5: Lumbar Puncture** (optional)

**APPENDIX 2: Sample size**

The sample size was determined from a power analysis of our pilot data that used similar proximal outcome measures. In the pilot data, medium effect sizes (.16-.34) were found for the Time (3)-by-Intervention (3) interaction (see below). Depending on the measure, 43 to 117 participants in total are needed to detect a significant Group x Time interaction effect with .80 power and .025 alpha level.

*Power calculations for the interaction (alpha=5%, power=80%) based on pilot data*

|  | Observed effect size (f) | Size of the effect | N necessary for adequate power per group |
| --- | --- | --- | --- |
| Delayed list recall | .179 | Medium  (correlation=0.7) | 43 |
| Face-name association | .160 | Medium  (correlation=0.3) | 117 |

**APPENDIX 3: Dissemination plan**

*Effect of leisure activities and cognitive training in persons with subjective complaint: the ENGAGE study.*

This paper that will report the effect on the outcomes measures for the first post-training assessment.

*Long-term efficacy of a combined leisure and cognitive training program in older adults with subjective cognitive impairment.*

This paper will report the data from the long-term follow-up.

*The efficacy of a new program using leisure activities and cognitive training to promote cognitive reserve in persons with subjective cognitive impairment: indicators of efficacy.*

This paper will focus on individual factors that moderate the efficacy of the training. In particular, one possibility is to look more carefully at « brain reserve » and cognitive reserve as moderators of efficacy.

*Increasing brain reserve through leisure and cognitive training: effect on patterns of brain activation.*

This paper will report the short-term effect (Pre vs first post-training assessment) of training on task-related activation.

*Increasing brain reserve through leisure and cognitive training: effect on brain connectivity.*

This paper will report the short-term effect (Pre vs first post-training assessment) of training on connectivity.

*Increasing brain reserve through leisure and cognitive training: effect on brain volume.*

This paper will report the short-term effect (Pre vs first post-training assessment) of training on regional brain volume including hippocampal volume.

*Maintenance of brain changes following cognitive training in persons with SCI*

This paper will look at the long-term (second post-training) maintenance of the brain changes by combining the different brain imaging outcomes (as there will probably be less power on this long-term assessment)

*The role of cognitive reserve and lifestyle in modulating the relation between brain atrophy and increased cognitive symptoms in persons with subjective complaints.*

This paper will analyse some of the pre-training data from the cohort of SCI. It will look at the relationship between indicators of cognitive reserve, brain atrophy and cognitive symptoms. Sample size could be increased by combining all SCI from CCNA.

*Patterns of task related activation in SCI and relation to structural changes.*

This paper will analyse some of the pre-training data from the cohort of SCI. It will look at the pattern of task-related activation in persons with of SCI and relate this pattern to regions of brain atrophy.

*Comparing the efficiency of two stimulating leisure activities: learning music versus learning a second language*

This paper will investigate subtler differences between our two stimulating leisure activities: learning music and learning Spanish as a second language. Our aim will be to identify similarities and specificities in training-related benefits following different types of activities.

**APPENDIX 4: Consent form of the ENGAGE study (participant’s version)**

| **Head of Research Project:** | - **Sylvie Belleville** Ph.D., Researcher at the Center of Research, IUGM. |
| --- | --- |
| **Co-Researchers on Site:** | - Ana Ines Ansaldo, Ph.D., Speech therapist, Researcher at the at the Center of Research, IUGM. - Louis Bherer, Ph.D., Researcher, IUGM and PERFORM. - Nathalie Bier, Ph.D., Occupational therapist, Researcher at the Center of Research, IUGM. - Marie-Andrée Bruneau, M.D., Psychiatrist, Researcher at the at the Center of Research, IUGM. - Patricia Da Cunha Belchior, Ph.D., Researcher at the at the Center of Research, IUGM. - Brigitte Gilbert, Ph.D., Neuropsychologist IUGM. |
| **Granting Organization:** | **Canadian Institutes of Health Research (CIHR)** |
| **Main Location of the Research Project:** | **Institut Universitaire de Gériatrie de Montréal** |

**INFORMED CONSENT TO ACT AS A PARTICIPANT IN THE STUDY:**

***COGNITIVE TRAINING PROGRAM FOR OLDER ADULTS: THE ENGAGE PROJECT***

This consent form describes what you may expect if you decide to participate in the research study: “Cognitive Training Program for Older Adults: the ENGAGE Project”. You are encouraged to read this consent form closely, and to ask the person who presents it any further questions you may have before making your decision whether or not to participate. This study is associated with the “Comprehensive Assessment of Neurodegeneration and Dementia (COMPASS-ND) Study”, which you have agreed to participate in. Only participants enrolled in the COMPASS-ND study can participate in the following study. The COMPASS-ND study is a pan-Canadian research study designed to assess individuals with different sorts of cognitive and motor changes seen in older adults. Both studies have been approved by the Research Ethics Committee of the Jewish General Hospital. They are sponsored by the Canadian Consortium on Neurodegeneration in Aging (CCNA), through a grant organized by the Canadian Institutes of Health Research (CIHR) and funded by multiple partners (CIHR, Alzheimer Society of Canada, Sanofi, New Brunswick Health Research Foundation, Robin and Barry Picov Family Foundation, Saskatchewan Health Research Foundation, Women’s Brain Health Initiative, Michael Smith Foundation for Health research, Alzheimer’s Research UK, Alberta Prion Research Institute, Nova Scotia Health Research Foundation and the Canadian Nurses Foundation).

**STUDY GOAL**

Previous studies demonstrated that programs aiming to train brain functions, such as memory and attention, called cognitive training interventions have had a positive impact on the participants’ memory and well-being. This purpose of this study is to compare the effectiveness of different cognitive training programs on older adults with memory concerns. These programs combine memory and attention training with stimulating leisure activities. For the purpose of this study, we aim to recruit 180 participants, men and women, aged between 60 to 85 years old, who have memory concerns, and who are enrolled in the COMPASS-ND study.

You (the research participant) are being asked to participate in this study because you have concerns about your memory. We will assess the impact that one of these training programs has on your memory, your reasoning, your daily functioning, and on brain imaging measurements. To participate, you will be required to have someone (partner, friend, relative) called a “study partner”, who will answer two questionnaires concerning your daily life activities and your mood.

**DESCRIPTION OF STUDY PROCEDURES**

This study will be held at the Research center of the IUGM (CRIUGM), 4545 chemin Queen Mary, Montréal H3W 1W4, or a partnered community centre, following your participation in the COMPASS-ND project.

Your participation includes up to **27 visits of an hour and a half (1h30) to three hours (3h)** over a **seven-month period** and **one (1) additional visit** **in two years**. During the study, you will be asked to:

- Provide information on your health, mood, daily life activities, memory, and hobbies;
- Undergo tests of memory, attention, reasoning, and daily function;
- Undergo a Magnetic Resonance Imaging (MRI) scans of your brain during which you will perform a memory task;
- Participate in a cognitive training intervention (primarily focused on memory and attention);
- Complete homework related to the program;
- Play videogames on a tablet, which will be lent to you for the duration of the study;
- Consent to having the collected data from the COMPASS-ND study shared with the ENGAGE study in a confidential manner.

**Description of the cognitive training programs:**

You will undergo one of the three cognitive training programs that are tested in this study. These programs contain educational sessions about the brain, memory, and attention, but they differ in terms of the main leisure activity offered:

- The ENGAGE-MUSIC program includes musical classes for beginners;
- The ENGAGE-SPANISH program includes Spanish classes for beginners;
- And the ENGAGE-DISCOVERY program provides knowledge training through the viewing of documentaries.

You can exclude either the MUSIC or SPANISH activity if you dislike one of them. You will then be randomly allocated to one of the remaining groups. All the groups will receive the same amount of training, over the same period of time (4 months). All the programs offer the same balance between educational and leisure activities.

All the training sessions will be held at the Research center of the IUGM or in a partnered community center, in groups of 5 to 8 participants:

- Twice a week for the first two months of the program;
- Then once a week for the last two months;
- For a total of twenty-four (24) sessions.

The sessions can last 2 to 2.5 hours. Between sessions, you will be given homework to apply concepts you learned in class. Some of the homework will be done on a tablet that will be lent to you for the duration of the study. To participate in these programs, you will need to have a wireless connection (Wi-Fi) at home. Training sessions may be audio recorded or videotaped for quality control purposes. The recordings will be accessed by the study coordinator who will assess the quality of the sessions and they will be deleted at the conclusion of the study. In addition, with your consent, these recordings may be used for research, teaching, or scientific conference purposes. You will not be identified by name. In these instances, the recordings will be kept after the conclusion of the program (maximum of 25 years after the end of the study).

**Evaluation sessions description:**

A total of **three (3) evaluation sessions of your cognitive function will occur in the following order**:

- A 2h session taking place 1 to 8 weeks before the start of the training;
- A 3h session taking place during the month following the end of the training;
- A 2h session taking place 2 years form now.

During those sessions, you will undergo a series of tests to evaluate different cognitive functions, such as your memory, attention, reasoning, and daily functioning. You will be asked to complete a number of computer-based, paper-and-pencil tasks and to complete some questionnaires. You will be asked to complete a portion of these questionnaires at home for approximately an hour. We may ask permission for the sessions to be watched, filmed, or recorded for training and evaluation purposes of the psychometrist. The recordings will only be accessible to the study coordinator, and will be deleted once they have been reviewed or at the conclusion of the study. If you agree to be recorded for psychometrist training purposes, we will ask you to consent to this at the end of this form.

We will ask your study partner to answer two questionnaires. First, we will ask your study partner to provide information on your activities of daily living (e.g., managing finances, getting groceries, taking medication, preparing a meal, remembering appointments, participation in hobbies, organizing activities, etc.). The second questionnaire will inquire about your mood and interest in social engagement.

Some of these tasks will be administered to you during your visits for the COMPASS-ND project. If you are already taking part in the CIMA-Q project, you may already have completed some of these tests. In both cases, to avoid repeating the same tests and questionnaires, we will ask you to consent to having the results of these neuropsychological evaluations shared with the ENGAGE research study. The results will be stored in the LORIS database (see details below) without identifying information.

**Magnetic Resonance Imaging (MRI) exam:**

A sub-group of participants will undergo a functional magnetic resonance imaging (fMRI) scan before and after the intervention. The first fMRI scan will take place during one of the COMPASS-ND visits. The second fMRI scan will occur after the conclusion of the intervention, and will be an additional 2H30 session. These visits will take place either at the Functioning Neuroimaging Unit of the IUGM, on 4545 Chemin Queen Mary, Montreal, QC, H3W 1W4, or at the Montreal Neurological Institute (MNI), on 3801 Rue University, Montreal, QC, H3A 2B4.

Magnetic Resonance Imaging gives images of the body and brain. *Functional* Magnetic Resonance Imaging enables us to understand the brain’s functioning, by showing the regions of the brain that become active when a person is asked to perform a specific task. When the person performs the task, there’s an increase in the blood flow to the part of the brain that is used during the activity. The blood flow to that region evokes a change in the magnetic signal transmitted by the brain, and this change can be detected by the scanner.

No substances will be injected for this procedure. You will lie down on a bed that will slowly be slid into a large tube. This tube is opened at both ends. An intercom system will allow you to communicate with the technician, if needed. For your comfort, we will ask you to wear either headphones or protective earplugs, to minimize the loud noises emitted by the machine. While the machine is functioning, it is important to stay still. A pillow will be put around your head to ensure that you are still. During certain time points of the MRI session, we will ask you to undergo a memory task, which will consist of watching a screen with images. We will ask you to memorize those images and their position on the screen. Once the MRI session is over, you will be brought to a quiet office, and we will ask you to recall the images and their positions.

The MRI session will last around 60 minutes. The whole visit, which includes the MRI scan, explanation of the procedure, and the memory task, will last around two and a half hours to three hours.

**Summary of the ENGAGE project visits:**

In summary, **when you have completed your 3^rd^ or 4^th^ visit for the COMPASS-ND project, you will meet with ENGAGE project team members for 28 visits in the following order**:

- - 1 neuropsychological evaluation session before the intervention at the CRIUGM.
  - 24 visits for the ENGAGE intervention, at the CRIUGM or at a partnered community center.
  - 1 neuropsychological evaluation session after the intervention at the CRIUGM.
  - 1 fMRI scan after the intervention at the MNI or CRIUGM.
  - 1 neuropsychological evaluation session 2 years after the beginning of the study at the CRIUGM.

The **total duration of your participation** in our study will be approximately **2 years**. If, in this time interval, due to changes in your health, you or your family decide that someone else should be responsible for making decisions for you on your behalf, we ask that the person who will be responsible for making these decisions sign a consent form stating that they agree to your continued participation in this study.

**INCIDENTAL FINDINGS**

Occasionally, an unexpected finding comes up in the course of assessing the participant, which may require further medical attention (for example, answers to a questionnaire suggesting sleep troubles or low mood). In addition, although the images from the MRI scan are not examined by a physician for diagnostic purposes, the team may come across a medically significant finding, which are called ‘incidental findings’.

As outlined by the Canadian Consortium on Neurodegeneration in Aging (CCNA) policy, all medically significant findings will be transmitted via the data management system to the study site staff where the participant was seen. The study site physician will be responsible for determining the significance of the finding. If the finding is judged medically significant, you will be contacted by the study physician or by your family doctor to arrange a visit and discuss the finding, along with possible treatment options. The finding and its follow-up will be documented in your research file and its outcome will be monitored until it has been resolved, or as long as you remain in the study. No data related to the research study will be put in your medical file.

**RISKS AND DISADVENTAGES ASSOCIATED WITH THE RESEARCH PROJECT**

Your participation in this study may involve some risks or discomforts, which are outlined below.

- **Magnetic Resonance Imaging**

To date, there are no known risks for undergoing an MRI scan if you do not have any of the contraindications for undergoing this type of scan. The MRI does not use any ionising rays (rays that can alter chemical or biological compounds) or any form of cancer causing agents.

Due to the strength of the magnetic field created by the machine, it is necessary to take certain precautions. There is a risk of injury if metal is introduced in the imaging room. The metal could be attracted by the magnet from the MRI. People with pacemakers, medical staples, artificial cardiac valves, cochlear implants, or metal objects in eyes are not permitted to undergo MRI studies. Before undergoing an MRI, we will ask you to fill a detailed questionnaire to determine if you have any of these contraindications. This questionnaire will reviewed by the MRI technologist.

In addition, an MRI can be anxiety provoking for some people due to loud clacking sounds produced by the scanner and the confined space of the tube. You may also experience discomfort as a result of having to stay still inside the scanner.

- **Cognitive training program**

You may experience fatigue due to the number of visits to the CRIUGM during the training program.

- **Evaluations**

You may experience mild fatigue due to the number of different assessments that will evaluate your memory and your attention. Performance anxiety can also be experienced by some participants. The assessments on mood and mental state can also lead to frustration, boredom, or fatigue. Certain questions can elicit negative emotions or memories. If you feel discomfort, at any moment during the assessments, inform the study personnel and they will stop the assessment. The study personnel will be available to you until you feel better and will then consult with you to see whether you want to continue the evaluation. Whether you wish to continue, stop for the day, or stop the entire assessment, the study personnel are obligated to comply with your wishes. If you feel distraught while filling the questionnaires at home, put the document(s) and take a break. If you are unable to complete the questionnaires, return them back to the research personnel and inform them of what happened. Whether or not you choose to continue the assessment, your decision will be respected.

**BENEFITS ASSOCIATED WITH THE RESEARCH PROJECT**

It is possible that you will personally benefit from your participation in this research study, but it cannot be guaranteed. The information gained from this research study may be used in the future to help people with age-related cognitive decline.

**VOLUNTARY PARTICIPATION AND WITHDRAWAL POSSIBILITY**

Your participation in this study is entirely voluntary. You have the right to refuse to participate or may discontinue participation at any time without jeopardy to the medical care that you, your study partner or family members receive at this institution. To do so, please notify the principal investigator, Dr. Sylvie Belleville, or one of her research personnel. The principal investigator, the Ethics Committee, or the Canadian Institutes of Health Research may decide to terminate your study participation, without your consent, if new information emerges that suggests that participation in this study may not benefit you, if you do not abide by the rules of the research project, or if there are administrative reasons to abandon the project. If you are withdrawn from the study for any reason, we will ensure that all your questions are answered prior to discontinuing your study participation. You can ask that your unused data be destroyed. However, the data that was already shared and stored in the LORIS database will remain.

You can also withdraw your consent to share and use your personal health information. If you choose to withdraw your consent, you must inform Dr. Sylvie Belleville. The information collected before your release of the study may still be used to preserve the study’s scientific integrity.

**NEW INFORMATION**

You will be immediately notified of any new findings that could influence your choice to participate in this study. You will have the opportunity to decide if you would like to continue your participation.

**DATA STORAGE AND FUTURE USE**

All data in this study will be used by the COMPASS-ND study and be stored in the Longitudinal Online Research and Imaging System (LORIS, a controlled access database at McGill University that meets international security and safety standards. Numerous safeguards are in place to keep your information confidential, including:

- Personal identifiers will be removed (i.e., name, date of birth, etc.);
- Your personal details will be kept separate;
- Your data will be attached to a random series of 6 numbers which will be how it is identified within the study;
- Stringent security measures will prevent unauthorized access or misuse.

These safeguards make it difficult to know which personal information came from you or another participant. However, we cannot guarantee that you will never be re-identified. In the event of a problem with privacy, the site investigator, or his/her delegate will notify you immediately. Only coded data, which does not include anything that might directly identify you, will be shared for study purposes.

**STORAGE AND FUTURE USE OF MRI IMAGES**

Your MRI scans will be stored in the McGill LORIS database. Your imaging data will be de-identified to protect your identity. All identifying information will be removed from the data before being shared. Only coded data, that does not have identifying information, will be shared for study purposes.

**DATA SHARING WITH THE COMPASS-ND PROJECT**

All collected data will be stored in the COMPASS-ND study database, and will comply with the sharing data rules elaborated by the CCNA (the project’s sponsor). The study’s data will be shared with other researchers worldwide, and will be used to advance future biomedical research projects that have received approval by the ethics committee. Such projects can take place in universities, hospitals, non-profitable organizations, businesses, or governmental laboratories. All researchers must follow the laws and ethical guidelines for biomedical research. To gain access to the data collected in this study, the researchers must agree to comply with the CCNA’s Publication and Data Access Policy.

**CONFIDENTIALITY**

While you take part in this study, the site investigator and his/her team will collect and take down information about you in a research study file. Only information necessary for the study will be collected. The information in your study file could include your past and present medical history, information about your way of life and test results from the exams and procedures done during this study. Your file could also contain other information, such as your name, your sex, date of birth, and your ethnicity. Additionally, through your study partner, we will gather information on how you perform daily life activities (e.g., if you have challenges going grocery shopping, or managing your finances, etc.) and your mood. All the information collected from you and your study partner will remain confidential, as stated by the law. Laws from Quebec and Canada will be respected.

To protect your privacy, your information will be identified by (coded) numbers or letters. Only the site investigator, and his/her team responsible for the COMPASS-ND study, can identify the code associated with your name. The study will use the information collected about you for research purposes, only to reach the study goals explained in this consent form. All information related to this study will be kept by the study’s principal investigator for 25 years after the conclusion of this study. The study information could be printed in medical journals or shared with other researchers at scientific meetings, but only in a manner where you will not be identified. To make sure the study is being conducted properly, your study file as well as your medical files could be checked by the study’s doctor, the study staff, a person authorized by the Research Ethics Committee of the Hospital, and the study’s sponsor. These persons and groups are obliged to respect your privacy.

You have the right to access your study file in order to check that the information gathered about you and to correct it, if necessary, for as long as the study researcher or the study site keeps this information (25 years after the completion of this study). However, you may only have access to certain information once the study has ended, so that the quality of the research study is protected.

The audio or video files recorded during the training sessions will be kept during the entire duration of the study for quality control purposes. They will be stored in the study coordinator’s computer, and these recordings will be password protected, under the responsibility of the principal investigator. Furthermore, if you gave your consent, audio or video recordings of training sessions will be kept for up to 25 years after the conclusion of the study for the purposes of studying, teaching, research, or scientific conferences.

Information and datasets are stored in the LORIS database under strict security provisions. Data submitted to any database must be coded, meaning it will not include anything that might directly identify you. There is a slight risk that there could be a breach in the security of this database system resulting in the access of information. Safeguards are in place to minimize the risk. The data are stored in the database to share with other qualified researchers.

**COMPENSATION**

You will receive $20/visit as compensation for transportation (taxi or parking) during the evaluation sessions, for a total of $60. You will also receive $30 for each Magnetic Resonance Imaging session. Note that you will not receive any financial compensation when you will attend the cognitive training program. However, this workshop will be issued free of charge, you will not have to pay to participate.

**COMMERCIALIZATION POSSIBILITY**

Your participation in this study could lead to the making of commercial products. However, you will not receive any money from the sales of these products.

**COMPENSATION IN CASE OF INJURY AND PARTICIPANT’S RIGHTS**

Should you suffer harm of any kind following any procedure related to the research study, you will receive the appropriate care and services, without additional cost. Unless stated by the law, you will not be compensated for salary loss, invalidity, or discomfort from prejudice. By agreeing to participate in this research study, you do not give up your legal rights (including the right to ask for compensation due to harm resulting from your participation) nor discharging the sponsor, the institution and the researchers of their civil and professional responsibilities.

**MEDICAL EMERGENCY AND PROCEDURES**

Please note that the IUGM is not an acute care hospital center that offers emergency care nor does it have doctors on site around the clock. Therefore, in the event of a medical emergency that would require immediate care, first aid will be given by on-site staff and you will be transferred to a nearby hospital.

**RESEARCH PROJECT FUNDING**

The principal investigator of this study project has received funding from the CIHR to conduct it.

**CONTACT PERSONS**

If you have any questions concerning the research project, or if you feel that you have encountered a problem as a result of your participation in this study, please contact the principal investigator, **Sylvie Belleville**, at the following number: **(514) 340 3540 #4767**, or the study coordinator **Aline Moussard**, at **(514) 340 3540 #3431**.

For all questions concerning your rights during your participation in this study, or if you have any complaints or comments regarding your experience in taking part in this research study, you can contact the **Local Commissioner of Complaints and Quality of Service of the CIUSSS Centre-Ouest-de-l’Île-de-Montréal or the ombudsman of the institution at (514) 340-8222, ex. 24222**.

**INFORMED CONSENT TO**

**ACT AS A RESEARCH AID IN THE STUDY CASE OF:**

***COGNITIVE TRAINING PROGRAM FOR OLDER ADULTS: THE ENGAGE PROJECT***

**CONSENT DECLARATION**

I read (or a person read it to me) the study description. I have been informed of the risks and the potential advantages, and we have answered all of my questions with my satisfaction. A signed copy of this consent form will be given to me. My participation is voluntary and I can withdraw myself from this study at any moment, without giving a reason, and without the medical care that I receive being affected in the future. I do not renounce any of my legal rights by signing this consent form. I accept to participate in this study.

Also:

I accept to be **FILMED AND RECORDED** for training, monitoring the collection of psychometric data, or training quality evaluation **(Optional)**.

| **Yes  No** |  | **Initials** |
| --- | --- | --- |

I accept that the **AUDIO AND VIDEO RECORDINGS** be used for studying, teaching, researching, or scientific conferences purposes **(Optional)**.

| **Yes  No** |  | **Initials** |
| --- | --- | --- |

I accept that all my collected **DATA** concerning the ***Comprehensive Assessment of Neurodegeneration and Dementia (COMPASS-ND) Study* be SHARED with the current study.**

| **Yes  No** |  | **Initials** |
| --- | --- | --- |

If I also act as a participant’s partner in the Consortium study for the Early Identification of Alzheimer’s Disease in Quebec (CIMA-Q), I accept that **all of my collected DATA in this study be SHARED with the current study.**

| **Yes  No**  **Not Applicable** |  | **Initials** |
| --- | --- | --- |

I accept **to be CONTACTED FOR RELATED STUDIES (optional)**

| **Yes  No** |  | **Initials** |
| --- | --- | --- |

I accept that **all of my collected DATA be SHARED anonymously with international researchers for research projects that could take place in universities, hospitals, non-profit groups, private businesses, or governmental laboratories, and be used for study cases that have not been elaborated.**

| **Yes  No** |  | **Initials** |
| --- | --- | --- |

|  |  |  |  |  |
| --- | --- | --- | --- | --- |
| **Research participant** (in print) |  | **Signature** |  | **Date** |

|  |  |  |  |  |
| --- | --- | --- | --- | --- |
| **Person getting consent** (in print) |  | **Signature** |  | **Date** |

|  |  |  |  |  |
| --- | --- | --- | --- | --- |
| **Head of Research Project** (in print) |  | **Signature** |  | **Date** |

**APPENDIX 5: Consent form of the ENGAGE study (informant’s version)**

| **Head of Research Project:** | - **Sylvie Belleville** Ph.D., Researcher at the Center of Research, IUGM. |
| --- | --- |
| **Co-Researchers on Site:** | - Ana Ines Ansaldo, Ph.D., Speech therapist, Researcher at the at the Center of Research, IUGM. - Louis Bherer, Ph.D., Researcher, IUGM and PERFORM. - Nathalie Bier, Ph.D., Occupational therapist, Researcher at the Center of Research, IUGM. - Marie-Andrée Bruneau, M.D., Psychiatrist, Researcher at the at the Center of Research, IUGM. - Patricia Da Cunha Belchior, Ph.D., Researcher at the at the Center of Research, IUGM. - Brigitte Gilbert, Ph.D., Neuropsychologist IUGM. |
| **Granting Organization:** | **Canadian Institutes of Health Research (CIHR)** |
| **Main Location of the Research Project:** | **Institut Universitaire de Gériatrie de Montréal** |

### INFORMED CONSENT TO ACT AS A STUDY PARTNER IN THE STUDY:

***COGNITIVE TRAINING PROGRAM FOR OLDER ADULTS: THE ENGAGE PROJECT***

This consent form describes what you may expect if you decide to participate in the research study: “Cognitive Training Program for Older Adults: the ENGAGE project”. You are encouraged to read this consent form closely, and to ask the person who presents it any further questions you may have before making your decision whether or not to participate. This study is associated with the “Comprehensive Assessment of Neurodegeneration and Dementia (COMPASS-ND) Study”, which you have agreed to participate in. It is sponsored by the Canadian Consortium on Neurodegeneration in Aging (CCNA), through a grant organized by the Canadian Institutes of Health Research (CIHR) and funded by multiple partners (CIHR, Alzheimer Society of Canada, Sanofi, New Brunswick Health Research Foundation, Robin and Barry Picov Family Foundation, Saskatchewan Health Research Foundation, Women’s Brain Health Initiative, Michael Smith Foundation for Health research, Alzheimer’s Research UK, Alberta Prion Research Institute, Nova Scotia Health Research Foundation and the Canadian Nurses Foundation).

**STUDY GOAL**

Previous studies demonstrated that programs aiming to train brain functions, such as memory and attention, called cognitive training interventions, have had a positive impact on the participants’ memory and well-being. This purpose of this study is to compare the effectiveness of different cognitive training programs on older adults with memory concerns.

You (the study partner) are being asked to participate in this study as a partner, family member, or close friend to the study participant. Your participation will consist of answering two short questionnaires concerning the daily life activities and mood of your partner.

**DESCRIPTION OF STUDY PROCEDURES**

We will ask you to complete two questionnaires. In the first questionnaire, you will be asked to evaluate your partner’s ability to perform a range of daily life activities (for example, managing finances, getting groceries, taking their medication, preparing a meal, remembering appointments, participating in hobbies, organizing activities, etc.). In the second questionnaire, we will ask you to evaluate your partner’s interest in social engagement and ability to initiate conversations. Completing each questionnaire will take 5 to 10 minutes. You will have to complete them before the intervention program, right after the conclusion of the program, and finally two years after the start of the study.

**RISKS AND DISADVENTAGES ASSOCIATED WITH THE RESEARCH PROJECT**

There are no risks or inconveniences associated with this research project.

**BENEFITS ASSOCIATED WITH THE RESEARCH PROJECT**

There is no guarantee that you will personally benefit by participating in this research study. All obtained results will advance scientific knowledge for the future prevention of cognitive decline associated with age.

**VOLUNTARY PARTICIPATION AND WITHDRAWAL POSSIBILITY**

Your participation in this study is entirely voluntary. You have the right to refuse to participate or may discontinue participation at any time without jeopardy to the medical care that you or the study participant receive at this institution. To do so, please notify the principal investigator, Dr. Sylvie Belleville, or one of her research personnel. The principal investigator, the Ethics Committee, or the Canadian Institutes of Health Research may decide to terminate your study participation, without your consent, if new information emerges that suggests that your participation in this study may not benefit you, if you do not abide by the rules of the research project, or if there are administrative reasons to abandon the project. The information collected about you prior to your withdrawal from the study will be kept as is required by law.

**DATA STORAGE**

All data in this study will be used by the COMPASS-ND study and be stored in the Longitudinal Online Research and Imaging System (LORIS), a controlled access database at McGill University that meets international security and safety standards. Numerous safeguards are in place to keep your information confidential, including:

- Personal identifiers will be removed (i.e., name, date of birth, etc.);
- Your personal details will be kept separate;
- Your data will be attached to a random series of 6 numbers which will be how it is identified within the study;
- Stringent security measures will prevent unauthorized access or misuse.

These safeguards make it difficult to know which personal information came from you or another participant. However, we cannot guarantee that you will never be re-identified. In the event of a problem with privacy, the site investigator, or his/her delegate will notify you immediately. Only coded data, which does not include anything that might directly identify you, will be shared for study purposes.

**DATA SHARING WITH THE COMPASS-ND PROJECT**

All collected data will be stored in the COMPASS-ND study database, and will comply with the sharing data rules elaborated by the CCNA (the project’s sponsor). The study’s data will be shared with other researchers worldwide, and will be used to advance future biomedical research projects that have received approval by the ethics committee. Such projects can take place in universities, hospitals, non-profitable organizations, businesses, or governmental laboratories. All researchers must follow the laws and ethical guidelines for biomedical research. To gain access to the data collected in this study, the researchers must agree to comply with the CCNA’s Publication and Data Access Policy.

**CONFIDENTIALITY**

No personal information concerning yourself or your health status will be collected during your participation in this study as a study partner. The answers to the two questionnaires will be attached to the participant’s study file. However, we will ask for your consent to access personal information collected from the COMPASS-ND study, such as your relationship with the study participant and the frequency of interaction. All collected data from yourself and the study participant will remain confidential, as stated by the law. Laws from Quebec and Canada will be respected.

To protect your privacy, your information will be identified by (coded) numbers or letters. Only the site investigator, and his/her team responsible for the COMPASS-ND study, can identify the code associated with your name. The collected information related to your study partner will be used for research purposes only, only to reach the study goals explained in this consent form. All information related to this study will be kept by the study’s principal investigator for 25 years after the conclusion of this study. The study information could be printed in medical journals or shared with other researchers at scientific meetings, but only in a manner where you will not be identified.

You have the right to access your study file in order to check that the information gathered about you and to correct it, if necessary, for as long as the study researcher or the study site keeps this information (25 years after the completion of this study). However, you may only have access to certain information once the study has ended, so that the quality of the research study is protected.

Information and datasets are stored in the LORIS database under strict security provisions. Data submitted to any database must be coded, meaning it will not include anything that might directly identify you. There is a slight risk that there could be a breach in the security of this database system resulting in the access of information. Safeguards are in place to minimize the risk. The data are stored in the database to share with other qualified researchers.

**COMPENSATION**

You will not receive any compensation for filling out the two questionnaires.

**COMMERCIALIZATION POSSIBILITY**

Your participation in this study could lead to the making of commercial products. However, you will not receive any money from the sales of these products.

**COMPENSATION IN CASE OF INJURY AND PARTICIPANT’S RIGHTS**

Should you suffer harm of any kind following any procedure related to the research study, you will receive the appropriate care and services, without additional cost. Unless stated by the law, you will not be compensated for salary loss, invalidity, or discomfort from prejudice. By agreeing to participate in this research study, you do not give up your legal rights (including the right to ask for compensation due to harm resulting from your participation) nor discharging the sponsor, the institution and the researchers of their civil and professional responsibilities.

**RESEARCH PROJECT FUNDING**

The principal investigator of this study project has received funding from the CIHR to conduct it.

**CONTACT PERSONS**

If you have any questions concerning the research project, or if you feel that you have encountered a problem as a result of your participation in this study, please contact the principal investigator, **Sylvie Belleville**, at the following number: **(514) 340 3540 #4767**, or the study coordinator **Aline Moussard**, at **(514) 340 3540 #3431**.

For all questions concerning your rights during your participation in this study, or if you have any complaints or comments regarding your experience in taking part in this research study, you can contact the **Local Commissioner of Complaints and Quality of Service of the CIUSSS Centre-Ouest-de-l’Île-de-Montréal or the ombudsman of the institution at (514) 340-8222, ex. 24222**.

### INFORMED CONSENT TO

### ACT AS A STUDY PARTNER IN THE STUDY:

***COGNITIVE TRAINING PROGRAM FOR OLDER ADULTS: THE ENGAGE PROJECT***

**CONSENT DECLARATION**

I read (or a person read it to me) the study description. I have been informed of the risks and the potential advantages, and all of my questions have been answered with my satisfaction. A signed copy of this consent form will be given to me. My participation is voluntary and I can withdraw myself from this study at any moment, without giving a reason, and without the medical care that I receive being affected in the future. I do not renounce any of my legal rights by signing this consent form. I accept to participate in this study.

As well:

I accept that all my collected **DATA** concerning the ***Comprehensive Assessment of Neurodegeneration and Dementia (COMPASS-ND) Study* be SHARED with the current study.**

| **Yes  No** |  | **Initials** |
| --- | --- | --- |

If I also act as a participant’s partner in the study for the Early Identification of Alzheimer’s Disease in Quebec (CIMA-Q), I accept that **all of my collected DATA in this study be SHARED with the current study.**

| **Yes  No**  **Not Applicable** | **_________** | **Initials** |
| --- | --- | --- |

I accept **to be CONTACTED FOR RELATED STUDIES (optional)**

| **Yes  No** |  | **Initials** |
| --- | --- | --- |

I accept that **all of my collected DATA be SHARED anonymously with international researchers for research projects that could take place in universities, hospitals, non-profit groups, private businesses, or governmental laboratories, and be used for study cases that have not been elaborated.**

| **Yes  No** |  | **Initials** |
| --- | --- | --- |

|  |  |  |  |  |
| --- | --- | --- | --- | --- |
| **Study partner** (Please print) |  | **Signature** |  | **Date** |

|  |  |  |  |  |
| --- | --- | --- | --- | --- |
| **Person getting consent** (Please print) |  | **Signature** |  | **Date** |

|  |  |  |  |  |
| --- | --- | --- | --- | --- |
| **Head of Research Project** (Please print) |  | **Signature** |  | **Date** |
